# APDS treatment in Germany: decision criteria, costs and contribution of leniolisib to outcomes

**DOI:** 10.3389/fimmu.2026.1842815

**Published:** 2026-08-10

**Authors:** Ulrich Baumann, Marie-Céline Deau, Karsten Franke, Leif G. Hanitsch, Georgios Sogkas, Kirsten H. Herrmann, Ulrich Bosch dos Santos, Christof Minartz, Aljoscha S. Neubauer, Fabian Hauck

**Affiliations:** 1 Immunology Unit, Paediatric Pulmonology, Allergy and Neonatology, Hannover Medical School (MHH), Hannover, Germany; 2 Institute for Immunodeficiency, Medical Center – University of Freiburg, Faculty of Medicine, University of Freiburg, Freiburg, Germany; 3 Center for Chronic Immunodeficiency (CCI), Medical Center – University of Freiburg, Faculty of Medicine, University of Freiburg, Freiburg, Germany; 4 Institute of Clinical Immunology, St. Marien Hospital, Siegen, Germany; 5 Immunodeficiency Outpatient Clinic, Institute for Medical Immunology, Charité – Universitätsmedizin Berlin, Berlin, Germany; 6 Department of Rheumatology and Immunology, Hannover Medical School (MHH), Hannover, Germany; 7 Pharming, Leiden, Netherlands; 8 Institute for Health- and Pharmacoeconomics (IfGPh), Muenchen, Germany; 9 Department of Pediatrics, University Children’s Hospital Heidelberg – Mannheim, Ruprecht-Karls-Universität, Heidelberg, Germany

**Keywords:** activated PI3 kinase delta syndrome (APDS), European society for immunodeficiencies (ESID), inborn error of immunity (IEI), leniolisib, PI3Kδ inhibitor, therapeutic decision criteria

## Abstract

**Purpose:**

Activated PI3 Kinase Delta Syndrome (APDS) is a rare inborn error of immunity. To better classify the contribution of a potential new therapeutic precision agent, leniolisib, medically relevant decision criteria were assessed upon which an outcomes model was built.

**Methods:**

Two online surveys and two roundtable discussions with 6 medical and scientific experts were conducted to obtain and discuss clinical and cost components. Based on the discussion results, key decision outcomes were modelled in an outcomes model.

**Results:**

Key decision therapeutic criteria of physicians were reliably found in two rounds of Maximum Difference Scaling, with efficacy being most important, followed by safety, disease severity, and quality of life impact. Relevant clinical outcomes in APDS, that were considered well quantifiable, were frequency of infections, lymphoproliferation, bronchiectasis, lymphoma, and mortality. For these outcomes, relevant improvements versus current standard of care were estimated for the new therapeutic precision agent leniolisib. Expert assessments in the online surveys, which were carried out by the 6 participants independently, showed low variance across participants. Ratings resulted in a projected reduction of mortality by use of leniolisib of ~16% at an age of 40 years (95% CI: [9%; 22%]). This age is currently reached only by 65% of APDS patients. Other changes were expected for costs, immunoglobulin replacement, treatment of lymphoma and other malignancies, bronchiectasis, and advanced lung disease, as well as allogeneic hematopoietic cell transplantation (alloHCT).

**Conclusions:**

Decision criteria of physicians for treating APDS are well in line with other therapeutic areas, with efficacy considered to be most relevant. Based on currently available data on leniolisib therapy, mortality and several patient-relevant morbidity endpoints are expected to improve in future clinical practice in Germany. Leniolisib may significantly modify the course of disease in APDS patients.

## 1 Introduction

Activated PI3 Kinase Delta (PI3Kδ-) Syndrome (APDS) is a rare genetically defined inborn error of immunity (IEI), that was first recognized in 2013 (1–3). It affects less than 1–2 individuals per 1,000,000 worldwide and is associated with lifelong morbidity and premature mortality. APDS patients present – although the clinical presentation of APDS is highly variable – with both immunodeficiency and immunodysregulatory features including recurrent respiratory tract infections, bronchiectasis, herpesvirus infections, autoimmunity, non-neoplastic lymphoproliferation, and malignant lymphoma, as well as neurodevelopmental delay and growth retardation. Most disease manifestations have a pediatric onset (4–6).

Leniolisib – a potential new therapeutic precision agent – is an oral, targeted phosphoinositide 3-kinase delta (PI3Kδ) inhibitor that is indicated for the treatment of APDS and targets specifically the underlying biochemical pathophysiology of the condition. By reducing the production of phosphatidylinositol-3,4,5-triphosphate (PIP3) and inhibiting the subsequent recruitment and phosphorylation of a broad range of downstream messengers, leniolisib restores signaling homeostasis in the PI3Kδ pathway. Furthermore, it modifies the cellular and clinical disease progression of APDS. The mode of action of inhibiting PI3Kδ targets normalization of deficiencies and dysregulation of B- and T-cell populations (7–9).

## 2 Materials and methods

To evaluate the contribution of leniolisib in the management of APDS, medically relevant decision criteria in Germany were assessed. Online surveys and roundtable discussions were conducted for this purpose. Based on the discussion results, key decision outcomes were modelled in an outcomes model. The model focuses on decision criteria in the treatment of APDS and their medical and economic impact. For the present study, key decision outcomes were the primary focus to model disease impact – although cost data were also considered. The purpose of considering costs at this point was to illustrate the main costs of APDS treatment in order to convey the magnitude of the costs incurred.

Data on treatment of APDS was collected based on two online surveys and two roundtable discussions within an expert panel. First, the online surveys were conducted, and in the next step, the evaluated results were discussed with all participants. The expert panel consisted of six physicians and scientists from German academic and reference centers with extensive clinical and scientific expertise in Activated PI3K Delta Syndrome (APDS) and other inborn errors of immunity (IEI). Several participants are affiliated with specialized immunology centers involved in the diagnosis, treatment, long-term follow-up, and research of APDS patients in Germany. Experts were selected based on their recognized expertise in clinical immunology, APDS management, translational research, and experience with precision therapies in rare immune disorders. The participating experts are listed as co-authors of the present study, and their institutional affiliations are provided in detail in the author information section of the manuscript.

Maximum Difference Scaling (MaxDiff analysis) – a conjoint analysis method for ranking people’s preferences by asking them multiple times to choose the best and worst option from a group of statements (10) – was performed to determine value in the treatment of APDS. MaxDiff analysis was conducted in two online surveys and validated in roundtable discussions in November 2024 and March 2025. Criteria ranged from areas such as quality of life and efficacy of therapy to costs. In a second online survey additional explanations for “efficacy of therapy” and “therapeutic impact” were given to the participants, as questions have arisen about the difference between these two items during the first roundtable discussion. The surveys were performed with dedicated software support (surveyking.com) and considering sample size planning: 10 total attributes were shown with 5 attributes per set and repeated 3 times to optimize result validity while keeping the number of displayed sets practical. To determine value in the treatment of APDS, similar analyses using comparable methods have already been conducted in other countries, namely in Spain (11, 12), which were considered for establishing the items of the present MaxDiff analysis.

**Figure 1:**
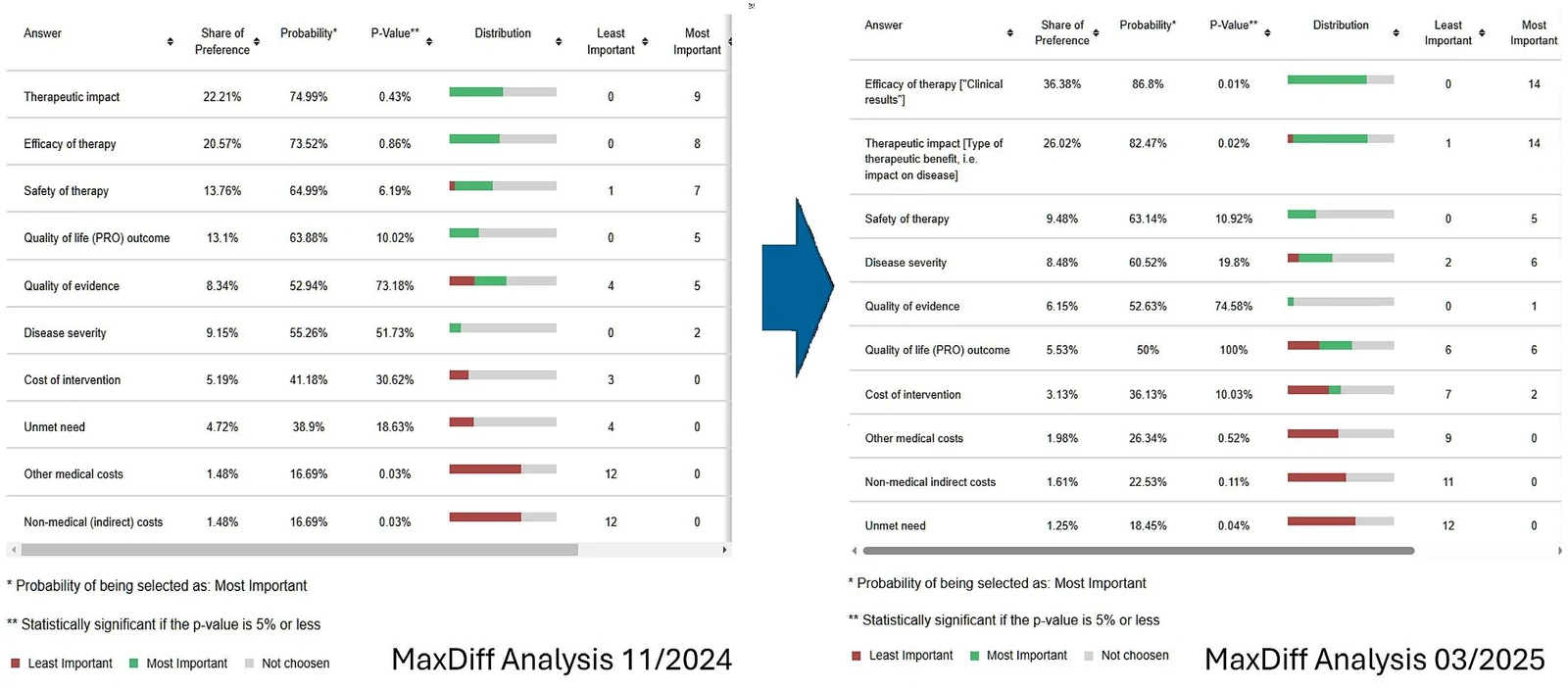
Determining value in the treatment of APDS in Germany. MaxDiff analysis: Results 11/2024 vs. 03/2025 (Counting Score for „most important”). Consistently and statistically significant, efficacy (“therapeutic impact” or “efficacy of therapy”) and safety were selected as most important items. Also, consistently and significant, “other medical costs” and “non-medical (indirect) costs” were rated as least important. “Unmet need” also ranked low, in the second survey reaching statistical significance. PRO, Patient-Reported Outcome.

For analysis, MaxDiff results were summarized using a counting score: this score was calculated as the frequency with which a criterion was selected as “most important” minus the frequency with which it was selected as “least important,” normalized across tasks. Higher scores indicate greater relative importance, allowing direct ranking of criteria (see **Figure 1**). In addition, selection frequencies were converted into choice probabilities by the software, and p-values were derived to assess whether criteria were chosen significantly more or less often than expected under equal preference. These statistics reflect the consistency and robustness of expert preferences rather than confirmatory hypothesis testing.

In addition to the value determination, the first online survey and roundtable discussion (11/2024) focused on costs and treatment patterns of APDS specifically for Germany. Comorbidities and their treatment were also considered. The medical experts gave their assessments of costs, reflecting the treatment patterns and procedures carried out for APDS patients in Germany. The most significant costs should be identified and quantified based on expert assessment reflecting German clinical practice and was supplemented by published literature where available. This was intended as an illustrative framework, because so far there has been a general lack of literature specifically addressing the healthcare costs associated with APDS, which is due to the rarity of the disease and is particularly true in Germany.

In the second online survey and subsequent roundtable discussion (03/2025), infections and other manifestations of APDS were compared between standard of care (SOC) and the treatment with leniolisib. In addition to infections, the following key manifestations were analyzed: non-neoplastic lymphoproliferation, cytopenia, gastrointestinal disorders (GI), bronchiectasis associated airway disease and advanced lung disease. Finally, considering the aforementioned disease manifestations, also mortality of APDS patients was investigated. Since there is currently no long-term data published on leniolisib, expert surveys should be used to estimate its most likely long-term effects on disease manifestations and mortality. Accordingly, using structured expert elicitation, the aim was to generate plausible estimates for the long-term impact of leniolisib on APDS manifestations and mortality. A structured questionnaire was developed for this purpose, which the experts were asked to complete based on their experience with leniolisib in Germany, which is available as an online **Supplementary Appendix 1**.

The SOC of treatment patterns and outcomes were judged by Kaplan-Meier data that were derived from individual patient data obtained from the APDS registry of the European Society for Immunodeficiencies (ESID) (13). This registry contains retrospective data from APDS patients and provides estimates for the occurrence of APDS manifestations for people treated with current SOC. Based on this data, the six participating medical experts were asked to provide estimates for upper and lower limits for the occurrence of manifestations and mortality for patients treated with current SOC. A lower plausible limit was defined as a value below which the expert considered the true value extremely unlikely to fall; the upper plausible limit was defined analogously. These elicited limits are plausibility bounds, not confidence limits. Lower and upper limits were summarized separately using the arithmetic mean and sample SD; they were neither transformed into 95% confidence intervals (CIs) nor discarded in favor of a variance-based interval. In the longitudinal plots, the SOC curves represent registry/literature Kaplan-Meier estimates. The experts also assessed the clinical plausibility of the fitted curves. Although all questions were mandatory, if the experts felt uncertain about their answer to a question, they had the option to indicate that accordingly in the questionnaire, resulting in censoring of the response. Contents and results of a UK cost-effectiveness model of leniolisib (14) were also taken into account, especially in the development process of the structure for the German model.

The experts were asked to provide estimates at different time points regarding patient age for each manifestation. Time points were age 12 - the lowest age for the anticipated leniolisib license in the European Union -, the age where the Kaplan-Meier data reached 100 (if applicable), and at least one other age where the Kaplan-Meier data varied from what would be clinically expected (e.g. a large drop in the curve). The maximum age for which data should be provided was 45 years. By considering different patient age, the questionnaire aimed to track the progression of APDS manifestations and mortality over the course of aging.

In the leniolisib part of the surveys, the six experts were then presented with the ESID APDS Kaplan-Meier data, representing current SOC, and were then asked to estimate the impact of leniolisib on the occurrence of the manifestations and mortality. Point estimates for the occurrence of manifestations were obtained in a 3- and 10-year timeframe (i.e. patient age 15 and 22 years) following the availability of leniolisib, given initiation of treatment at age 12. The 3- and 10-year estimates aim to capture both, medium and long-term benefits of leniolisib treatment. A duration of 3-years was selected for short-term estimates as stabilization was expected to be achieved at this time. Additionally, the experts were asked about the improvement (reduction of prevalence) and recurrence of certain manifestations upon treatment with leniolisib. Kaplan-Meier curves were determined based on the collected data and compared with the ESID data, thereby illustrating the effects of leniolisib compared to SOC. The 95% CIs were calculated for the expected occurrence of various manifestations as well as for mortality as indicators of variability. Two-sided 95% CIs around the arithmetic mean were calculated using Student’s t-distribution because the standard deviation was estimated from a small sample of six respondents. The CIs were calculated as $\bar{x}\pm t_{0,975,n-1}\cdot \frac{s}{\sqrt{n}}$. With (n=6) and five degrees of freedom, the corresponding critical value was 2.5706.

## 3 Results

### 3.1 Therapeutic decision criteria: MaxDiff analysis

The MaxDiff analysis showed that “efficacy of therapy” and “therapeutic impact” are ranked consistently in both analyses as the most important criteria to determine value of a new therapy for APDS patients in Germany (see **Figure 1**). “Safety of therapy” is also consistently seen as the second important criterion. “Quality of life”, “disease severity” and “quality of evidence” are in the middle range, with some minor changes in the two unaided analyses performed. Cost considerations scored clearly lower, reflecting a focus on clinical outcomes over financial aspects. “Unmet need” scored also low, showing its low to moderate importance for clinicians. Results of the second survey (03/2025) were well consistent to the first survey (11/2024), especially regarding the most important decision criteria. This confirms the reliability of the results: a (new) therapy primarily must be effective and safe. Of note, for the most relevant criteria also highly homogeneous results between the participating experts were recorded.

### 3.2 Key costs components of APDS treatment

Although costs are not the focus of the model, main cost drivers in the treatment of APDS patients in Germany were identified during the first roundtable discussion (11/2024). The purpose of the survey and discussion was to gain an overview of the most significant costs associated with the treatment of APDS patients. This was intended to provide a context for comparing the cost of treating this rare disease with that of other diseases. However, the goal was not to conduct a comprehensive cost survey, but rather to identify key cost areas and generate indicative figures for those areas. When considering costs in the model, a sickness funds’ perspective was taken, i.e., which expenses are incurred by sickness funds for the treatment of APDS patients. Immunoglobulin replacement, treatment of lymphoma and other malignancies, bronchiectasis, and advanced lung disease, as well as allogeneic hematopoietic cell transplantation (alloHCT) were important considerations. Obviously, the costs are very individual and depend on the patient’s condition and disease severity. Three key cost areas could be identified: immunoglobulin replacement therapy (IgRT), treatment of advanced lung disease and alloHCT (see **Table 1**).

**Table 1:** Key cost components in the treatment of APDS in Germany.

Description	Costs per year and patient [€]	Annotation
Immunoglobulin replacement therapy (IgRT)	~40,000	- depending on required dose - subcutaneous (SC) or intravenous (IV)
Treatment of advanced lung disease	up to 75,000	- depending on disease severity
Allogeneic hematopoietic cell transplantation (alloHCT)	150,000 - 250,000	- costs per alloHCT - depending on complexity of the individual patient’s case

IgRT was considered as a key contributor to costs of standard care of APDS. One reason for this is that it is one of the most commonly observed treatment associated with APDS alongside antibiotics and corticosteroids (15). The two modalities of IgRT, subcutaneous (SC) and intravenous (IV) application, also differ in their costs. While SC medication is more expensive than that of IV, the latter also requires presence of a physician incurring additional fees. After all, the costs substantially depend on the required dose. In addition, the required dose is related to disease manifestation (e.g. bronchiectasis). Historically, APDS treatments have targeted clinical manifestations, with approaches tailored to individual symptoms (15). This may increase the range of IgRT costs depending on individual symptoms, but according to experts’ estimates IgRT costs average about €40,000 annually per APDS patient in Germany. This assessment is also consistent with data from a US study, which reports average annual IgRT costs of $37,963 to $94,907 for the treatment of APDS patients (16).

Besides the treatment of lymphoma and other malignancies, bronchiectasis associated airway disease and advanced lung disease were considered as further important components of cost in the treatment of APDS patients: According to the experts, severe cases of advanced lung disease can generate treatment costs of up to €75,000 annually per patient. Above all, the costs depend on the severity of the disease and the associated hospital stays and necessary medication. With brensocatib, the European Medicines Agency (EMA) has approved a new drug in the EU that is used to treat bronchiectasis. Brensocatib is being marketed in the US, while its launch in Germany in 2026 was canceled shortly before the planned date for commercial reasons. Nevertheless this new therapy has the potential to become the standard of care for bronchiectasis, thereby also impacting future treatment outcomes and costs.

AlloHCT was assessed by the experts as the treatment with the highest costs. However, in most instances it is a once-in-lifetime intervention which carries a significant transplant-related mortality, but offers a potential immunologic cure for patients with APDS (17). That doesn’t mean, though, that treatment costs can be completely avoided after a successful alloHCT in the long run. It will not alleviate established structural lung damage such as bronchiectasis (18). Depending on the complexity of the individual patient’s case, expenses can reach up to €250,000 per transplantation procedure and follow-up care. Some patients need a second or third transplantation due to graft rejection or graft failure, which may result in relatively high total costs per patient. Though, international evaluations show that the frequency of a second (9.1%-22.2%) or third procedure (0%-1.8%) in APDS patients is rather low (17, 19, 20). The transplant procedure at the hospital accounts for the majority of the costs, at approximately €125,000 (21). In addition to the costs of the transplant procedure, the secondary costs of alloHCT are high, as patients often require intensive care after transplantation. Experience with alloHCT of APDS patients is relatively limited, which may lead to higher costs due to more complicated courses.

The cost profiles of alloHCT and leniolisib differ fundamentally. AlloHCT is a front-loaded, potentially curative intervention with substantial procedure-related mortality, follow-up costs, and possible retransplantation, whereas leniolisib is a chronic therapy with accumulating costs over the treatment lifetime. The present analysis did not model lifetime leniolisib costs, discounting, or downstream cost offsets and therefore does not constitute a comparative cost-effectiveness analysis.

### 3.3 Key APDS manifestations: SOC vs. leniolisib

**Figure 2:**
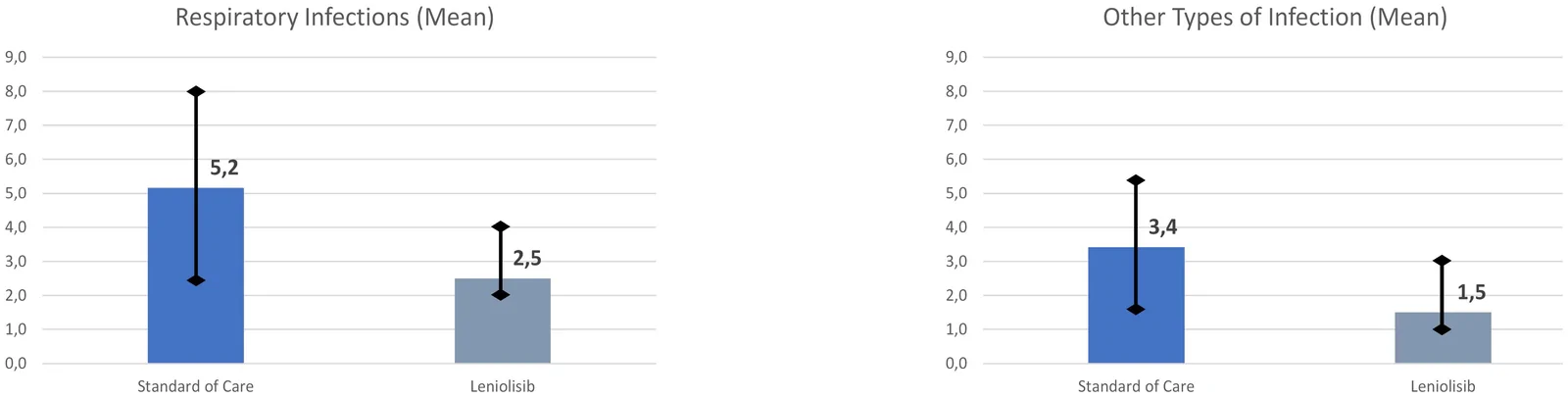
Number of infections per year (mean): SOC vs. leniolisib estimate.

APDS patients typically present with respiratory tract infections (RTI) and other types of infections (5, 22, 23). Recurrent infections can be managed with IgRT and antibiotic prophylaxis (5). Despite high-dose IgRT recurrent infections can be life-threatening and may require alloHCT. Therefore, it is crucial to minimize not only the number of infections but, more importantly, their severity. In this context, immunomodulatory therapies used to treat APDS should ideally avoid exerting additional immunosuppressive effects, as these could further exacerbate the underlying immunodeficiency and consequently increase the risk of severe infections According to the experts surveyed, the range of infections under SOC was estimated to be between 2.3 and 8.0 per year (average 5.2 per year for respiratory infections) or 1.5 and 5.3 per year (average 3.4 per year for other types of infections). Even if infections cannot be completely avoided, the assessment was that leniolisib can significantly reduce their number, especially in APDS1 patients, so that the lower limit is steadily reached. It was assumed that under leniolisib, the average number of infections per year is 2.5 (respiratory infections) or 1.5 (other types of infections; see **Figure 2**). According to the experts, leniolisib can potentially make an important contribution to keeping infections to a minimum and thus improving patients’ quality of life (7, 9, 24).

**Figure 3:**
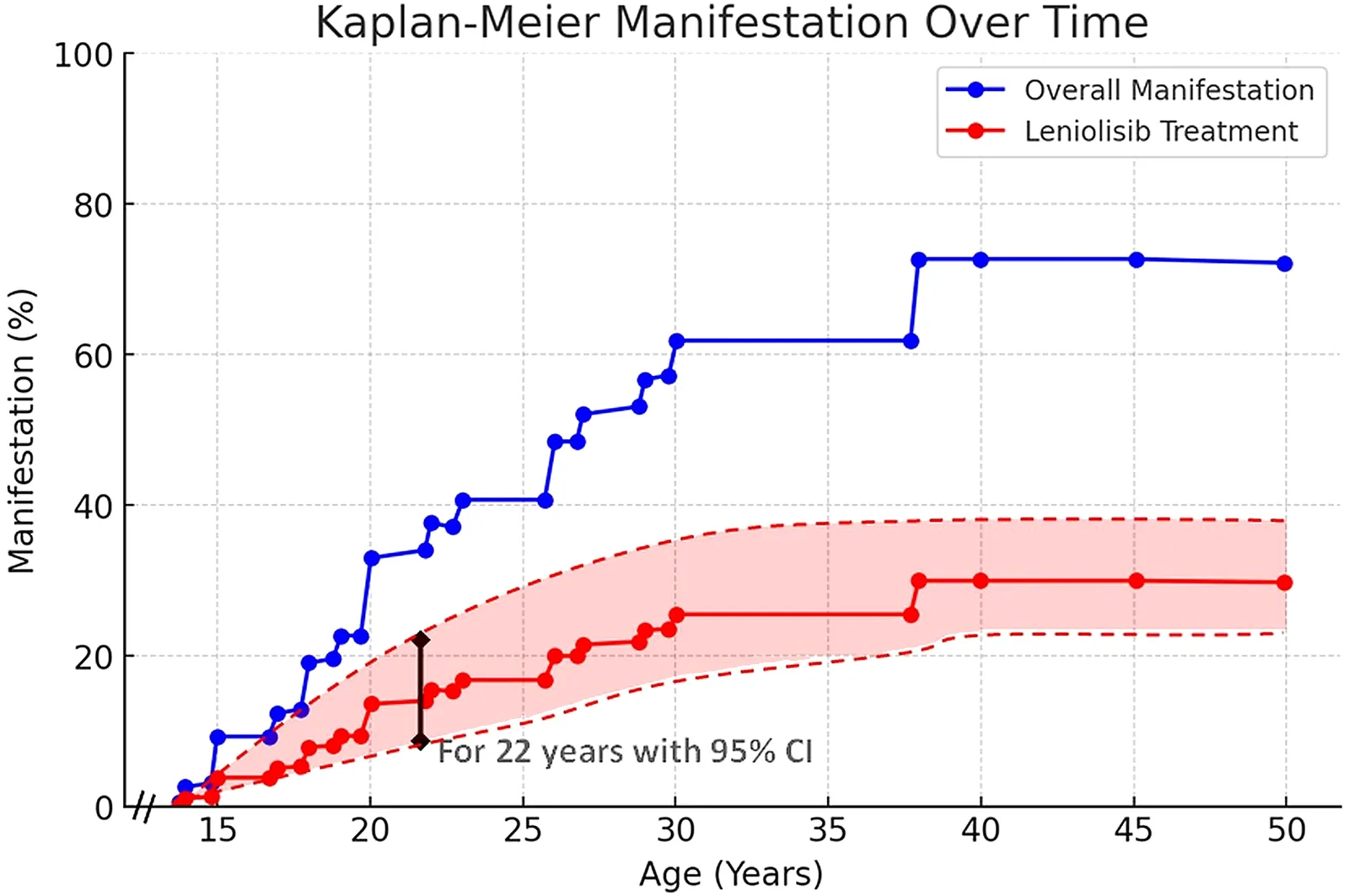
Lymphoproliferation: overall manifestation vs. leniolisib treatment. Inverted Kaplan-Meier data from ESID vs. German leniolisib estimate (mean 15.3% incl. 95% CI: 6.8–23.9% for age 22 years). ESID, European Society for Immunodeficiencies; CI, confidence interval.

Respiratory tract infections frequently coexist with non-neoplastic lymphoproliferation, including lymphadenopathy and splenomegaly. APDS is also associated with overt malignant lymphoma, a distinct endpoint that contributes to premature mortality (25). Immunosuppressive therapies and alloHCT aim at controlling lymphoproliferation and lymphoma, but APDS patients still have an unmet need for therapeutic options which address immunodeficiency and lymphoproliferation alike with limited adverse effects (5). Leniolisib, selectively targeting PI3Kδ mediated signaling, can reduce PI3Kδ constitutive pathway activation and decrease clinical lymphoproliferation – indicating that it may improve development, trafficking, and maintenance of B and T cells (8, 9). Accordingly, the experts projected fewer non-neoplastic lymphoproliferative manifestations under leniolisib (see **Figure 3**). However, reduction in bulk lymphadenopathy was not treated as a 1:1 surrogate for malignant transformation, and lymphoma risk was elicited and reported separately. Overt lymphoma was elicited separately from non-neoplastic lymphoproliferation. For patients without lymphoma at age 12, the SOC reference risks were 2.6% at 3 years and 10.5% at 10 years; corresponding leniolisib expert means were 1.5% (SD 0.9%) and 4.4% (SD 3.4%). For patients without lymphoma at age 20, SOC reference risks were 0.0% and 11.2%, compared with leniolisib means of 1.3% (SD 2.0%) and 5.3% (SD 4.7%). For patients recovered from lymphoma by age 25, no single recurrence point estimate was elicited; mean lower/upper plausible limits for recurrence within 10 years were 9.0%/21.7% under SOC and 3.2%/8.5% under leniolisib (see **Table 2**).

**Table 2:** Overt lymphoma risk and recurrence estimates.

Population	Horizon/estimate	SOC mean (%)	SOC SD (%)	Leniolisib mean (%)	Leniolisib SD (%)	n
No lymphoma at age 12	3-year risk	2.6	—	1.5	0.9	6
No lymphoma at age 12	10-year risk	10.5	—	4.4	3.4	6
No lymphoma at age 20	3-year risk	0.0	—	1.3	2.0	6
No lymphoma at age 20	10-year risk	11.2	—	5.3	4.7	6
Recovered by age 25	10-year recurrence L	9.0	7.4	3.2	3.8	6
Recovered by age 25	10-year recurrence U	21.7	11.3	8.5	9.0	6

Overt lymphoma was elicited separately from non-neoplastic lymphoproliferation. SOC 3- and 10-year risks are ESID registry-derived reference estimates. For recurrence after age 25, L/U denote lower/upper plausible limits; no single recurrence point estimate was elicited.

APDS is often associated with cytopenia due to various causes such as autoimmunity or concomitant infection (22). While studies have demonstrated a positive effect of leniolisib on cytopenia (5, 9), the experts surveyed were rather cautious in their assessments. The effect of leniolisib on cytopenia was considered rather low: For patients without cytopenia at age 12, the ESID-derived SOC risks presented to the panel were 3.3% at 3 years and 5.2% at 10 years; corresponding leniolisib expert means were 2.2% (SD 2.0%) and 4.3% (SD 3.4%). Similarly, the experts expected only limited impact on gastrointestinal disorders: For gastrointestinal manifestations, SOC risks were 7.1% and 14.3%, compared with leniolisib means of 5.4% (SD 1.6%) and 11.0% (SD 4.1%) (see **Table 3**). Significant improvement in gastrointestinal disorders was expected to become apparent later in time, when many other immunological manifestations have normalized. Among patients with an existing manifestation, the experts estimated 3-year improvement under leniolisib in 72.0% (SD 16.3%) for cytopenia and 47.7% (SD 27.2%) for gastrointestinal manifestations. These descriptive estimates indicate modest and heterogeneous projected prevention effects, with greater uncertainty for improvement in gastrointestinal disease.

**Table 3:** 3- and 10-year risks of cytopenia and gastrointestinal manifestations under SOC and leniolisib.

Manifestation	Horizon	SOC mean (%)	SOC SD	Leniolisib mean (%)	Leniolisib SD (%)	N
Cytopenia	3-year risk	3.3	—	2.2	2.0	6
Cytopenia	10-year risk	5.2	—	4.3	3.4	6
GI manifestations	3-year risk	7.1	—	5.4	1.6	6
GI manifestations	10-year risk	14.3	—	11.0	4.1	6

SOC values are ESID registry-derived point estimates presented to the experts and therefore have no expert-response SD. Leniolisib values are arithmetic mean and sample SD across the contributing experts. GI, gastrointestinal; SD, standard deviation; SOC, standard of care.

**Figure 4:**
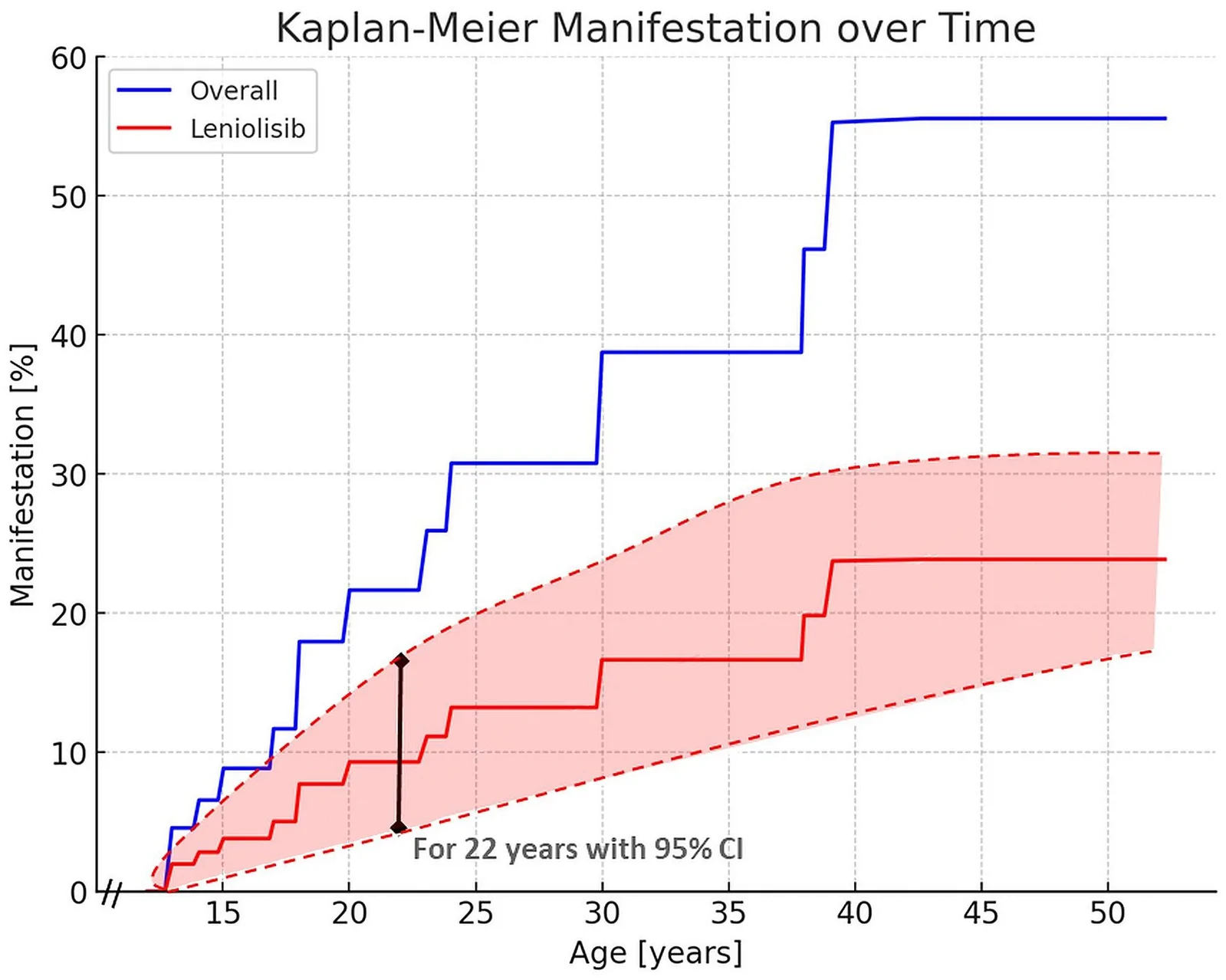
Bronchiectasis-associated airway disease: overall manifestation vs. leniolisib treatment. Inverted Kaplan-Meier data from ESID vs. German leniolisib estimate (mean 9.3% incl. 95% CI: 3.2–15.4% for age 22 years). ESID, European Society for Immunodeficiencies; CI, confidence interval.

Bronchiectasis is a common complication of lung infections in APDS patients. This irreversible lung damage further aggravates the problem of recurrent and chronic inflammatory lung disease and this significantly contributes to poor quality of life. In order to decelerate the progression of bronchiectasis and maintain optimal lung function and quality of life, it is imperative to establish an early diagnosis and implement an effective treatment plan (5). Especially reducing the frequency of respiratory infections may also reduce the risk of developing associated organ damage, such as bronchiectasis (24). As shown above, leniolisib has the potential to reduce the number of respiratory infections. Thus, it is expected based on expert projections to be very effective in slowing the development of bronchiectasis. The Kaplan-Meier curves generated from ESID data and expert assessments for SOC vs. leniolisib suggest that manifestations of bronchiectasis were expected to decrease significantly (see **Figure 4**). Being aware of the risks APDS patients face and initiating an effective treatment at an early stage may help limiting disease manifestations and maintaining quality of life. When precision therapy is available secondary damages to the lungs could be prevented (18).

**Figure 5:**
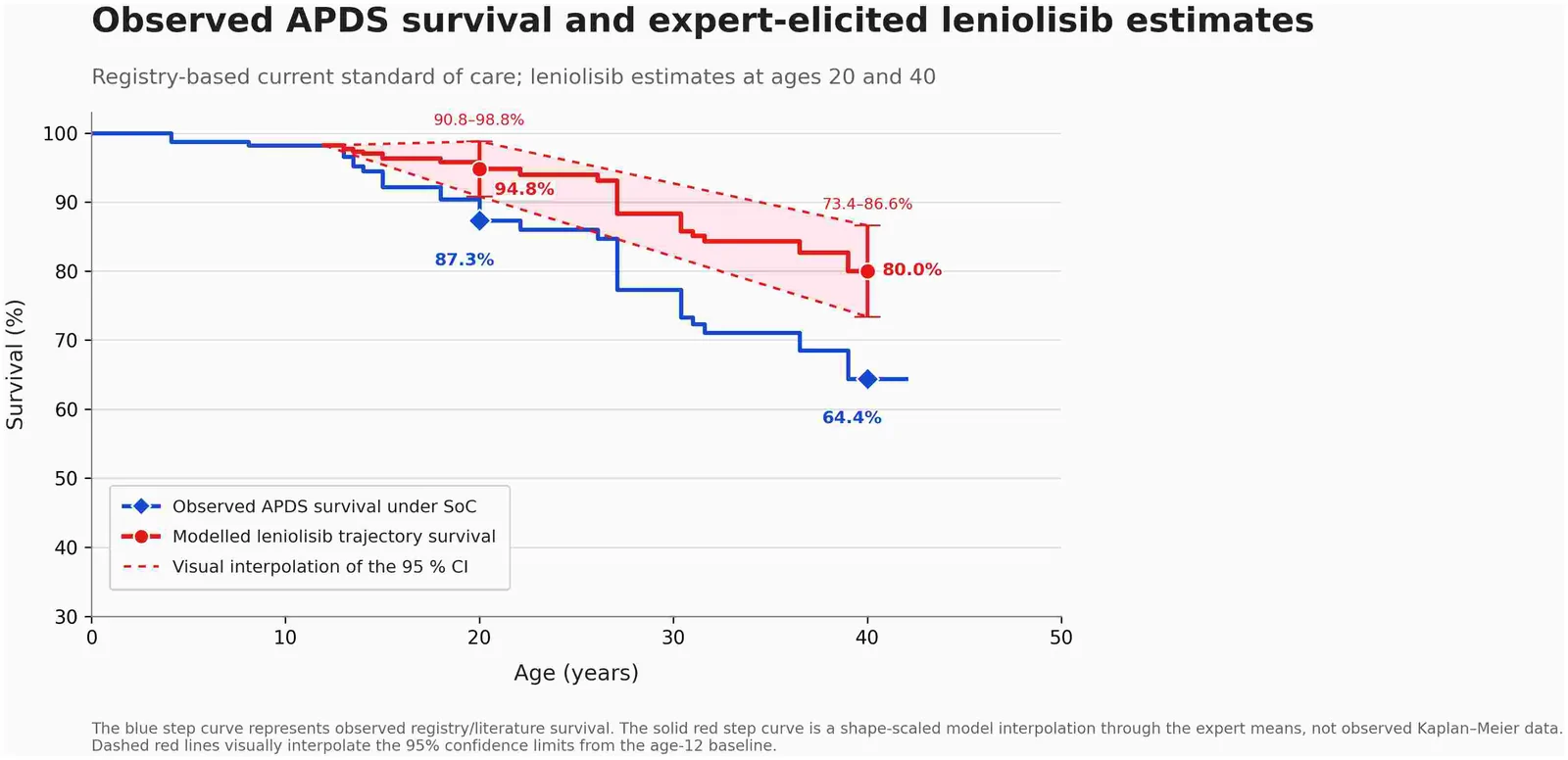
Mortality: APDS SOC vs. leniolisib treatment. Kaplan-Meier data from Hanson and Bonnen (26, 30) vs. German leniolisib estimate (incl. 95% CI for age 20 and 40 years). APDS, Activated PI3K delta syndrome; SOC, Standard of Care; CI, confidence interval.

These exploratory results have already shown that the experts expected leniolisib to have a beneficial effect, albeit to varying degrees, on manifestations of APDS and the patients’ quality of life. To date, no comparative clinical long-term experience with leniolisib is available, which is why the long-term effects cannot yet be substantiated with data from clinical studies – especially regarding mortality. Despite available SOC treatments, life expectancy for patients with APDS is reduced. A Kaplan-Meier survival analysis for APDS showed the conditional survival rate at the age of 20 years was 87%, age of 30 years was 74%, and ages of 40 and 50 years were 68% (26). The experts assumed that leniolisib can significantly increase the life expectancy: The model projected an absolute survival difference of 15.6 percentage points at age 40 (80.0% with leniolisib versus 64.4% under SOC). Treating the SOC estimate as a fixed reference, the 95% CI based solely on uncertainty in the elicited leniolisib mean was 9.0–22.2 percentage points. For leniolisib the estimated mean survival was 94.8% (SD: 3.8%; 95% CI: 90.8–98.8%) at age 20 years and 80.0% (SD: 6.3%; 95% CI: 73.4–86.6%) at age 40 years. The projected survival difference represents the modelled cumulative consequence of the assumed effects on APDS manifestations, compared with the currently observed survival trajectory shown in **Figure 5**. All aggregated expert-elicitation results are presented in an online **Supplementary Appendix 2**.

## 4 Discussion

This study assessed therapeutic decision criteria for Activated PI3 Kinase Delta Syndrome (APDS) in Germany and modelled the potential contribution of leniolisib to patient outcomes. Efficacy and safety were identified as the most important factors in decision-making, while costs played a less significant role. Expert opinion indicated that leniolisib could significantly reduce the risk of infection, lymphoproliferation, bronchiectasis and lymphoma, and improve survival rates compared to the current standard of care.

Regarding decision criteria, findings are consistent with international literature on APDS and, in general, for medical treatment prioritization. Previous multi-criteria decision analyses in Spain also found that therapeutic efficacy was the paramount determinant in evaluating new therapies, followed by safety and quality of life, whereas cost considerations were of lesser importance (11, 12). Our findings are consistent with those results. Similarly, German surveys of IEI centers have highlighted efficacy and safety as primary decision criteria when introducing novel therapies in rare immune disorders (25). A major difference to the Spanish results (11, 12) is, that “unmet need” was rated very low by the experts.

The obtained clinical projections of reduced infection frequency under leniolisib align very closely with controlled trial data reported to date. Rao and colleagues demonstrated that leniolisib significantly lowered infection burden and normalized immunoglobulin M levels, indicating a durable immunological benefit, and that infections reduced from mean >4 per year before leniolisib to 3 in year 1 of treatment and 0.2 per year in year 6 of treatment (7–9, 24) In our analysis, experts estimated a decrease in mean annual respiratory infections to approximately 2.5 episodes, consistent with the reductions reported in clinical studies for the first 2 years of treatment. Importantly, fewer infections were expected to contribute to prevention of bronchiectasis, a major driver of morbidity in APDS (5, 18).

Non-neoplastic lymphoproliferation and overt lymphoma are related but diagnostically distinct. Changes in lymph-node size under therapy are therefore not a validated surrogate for malignant potential. Recent inborn errors of immunity (IEI) data demonstrate substantial histologic heterogeneity, including reclassification of presumed lymphoma as reactive disease and germinal-center transformation patterns in non-neoplastic lymphoproliferation; serum immunoglobulin M (IgM) and circulating T-follicular-helper-cell findings may complement, but not replace, integrated clinical, genetic, virologic, and pathologic assessment (27). Lifelong surveillance remains warranted, including attention to Epstein-Barr Virus (EBV)/Cytomegalovirus (CMV) chronicity, serum IgM, lymphocyte phenotyping where available, and tissue reassessment when clinical or biomarker findings are suspicious or discordant.

A balanced pharmacological interpretation is also required. Preclinical studies with potent PI3Kδ inhibitors, including idelalisib and duvelisib, showed activation-induced cytidine deaminase (AID) overexpression and increased genomic instability in B cells (28). These findings cannot be directly extrapolated to leniolisib, and an APDS-focused clinical perspective found no established causal association between leniolisib and lymphoma while emphasizing the need for larger cohorts and long-term follow-up (29). Accordingly, any modeled lymphoma-risk reduction under leniolisib remains hypothesis-generating, and continued malignancy surveillance is necessary. While cytopenia and gastrointestinal manifestations were judged less responsive to leniolisib in the expert assessment, modest benefits cannot be excluded. Previous trial data demonstrated some improvement in hematologic abnormalities (9), but the effect appears heterogeneous and may depend on disease stage and co-morbid conditions.

Most importantly, our analysis projects an absolute survival difference of 15.6 percentage points at age 40. This model-based scenario projection should not be interpreted as a demonstrated survival benefit or causal treatment effect. It extrapolates from expert estimates and short-term surrogate outcomes across 28 years and is sensitive to unmeasured baseline disease damage, viral chronicity, malignancy risk, treatment exposure, and competing interventions. Nevertheless this projection is plausible given the cumulative impact on infections, risk of malignancy, and progressive lung disease. Survival analyses from registry data highlight the unmet need, with a reduced survival of 68% by the age of 40 under current SOC (14, 26). Our findings therefore putatively extend existing real-world evidence by quantifying the potential of precision therapy to modify disease course and life expectancy.

Overall, the German expert consensus suggests that leniolisib addresses the central pathophysiology of APDS and targets clinically meaningful outcomes. Despite the paucity of long-term real-world evidence, the available trial data and registry-based projections suggest that leniolisib has relevant potential in the management of APDS and may impact both morbidity and survival rates.

## 5 Limitations

This study has several limitations. First, it is important to note that the projections rely on structured expert judgement and modelling based on registry data, rather than long-term, real-world evidence with leniolisib in German APDS patients. While trial data and international registries provide a valuable foundation, assumptions concerning projected survival differences and long-term disease modification remain hypothetical and require validation in prospective studies. Expert opinions are prone to subjective bias, coincidental factors and technical errors, but offer added value in situations where the available research is still limited as in this case. In addition, expert knowledge takes into account the real-world evidence, whereas trials are often conducted in highly selected patient populations. While trials – especially randomized controlled trials – have high internal validity due to low bias from confounding factors, they often have low external validity if the study conditions do not reflect typical real-world conditions of medical practice. Expert assessments are therefore helpful in translating study results into real-world practice, in order to move from “clinical efficacy” to “community effectiveness”, but do have limitations.

Second, the number of participating experts was limited to six country-specific experts, which may restrict the generalizability of the findings, although the high consistency of responses suggests robustness. Third, the modelling approach is based on data from the European ESID registry and extrapolated to German practice; however, differences in local treatment pathways, costs, and patient characteristics may affect the transferability of the results. Fourth, the health-economic component was illustrative rather than a lifetime cost or cost-effectiveness model and thus cannot establish comparative economic value versus alloHCT or SOC.

Fifth, there are limitations of predictive modeling: In particular, the approximately 16-percentage-point age-40 survival difference is a scenario projection over 28 years from assumed treatment initiation at age 12. The model cannot separate treatment effects from mortality determinants independent of current PI3Kδ activity, including pre-existing structural lung damage, chronic or latent EBV/CMV infection, prior malignancy, age and accumulated organ damage, adherence, and the competing risks and potential benefits of alloHCT. These factors were not modeled at patient level; consequently, the analysis does not support causal or individualized survival predictions.

Finally, the study focused primarily on clinically and economically relevant endpoints. Given the rarity of APDS and the currently limited availability of long-term prospective and real-world data, particularly in Germany, the present study partly relied on structured expert elicitation and modelling approaches. Therefore, the projected effects of leniolisib on morbidity and mortality should be interpreted cautiously and regarded as exploratory rather than confirmatory. In addition, although leniolisib has demonstrated clinically meaningful benefits in APDS, limitations remain regarding the currently available duration of follow-up, the absence of extensive long-term safety data, and the uncertainty surrounding sustained disease modification in broader real-world patient populations. Further prospective clinical studies and long-term follow-up analyses will be required to validate the long-term clinical impact of leniolisib under routine clinical practice conditions.

## 6 Conclusions

Decision criteria of physicians for treating APDS are well in line with other therapeutic areas, with efficacy and safety considered to be most relevant. Based on data on leniolisib available today, mortality and several patient-relevant morbidity endpoints are expected to improve in future clinical practice in Germany. Leniolisib may also significantly modify the course of disease in APDS patients.

## Data Availability

The datasets presented in this article are not readily available because the expert assessments generated allow potential identification of individual persons (n=6). Requests to access the datasets should be directed to aljoscha.neubauer@ifgph-muenchen.de.

## Supplementary material

Appendix 1 Online questionnaire.

Appendix 2 Aggregated expert-elicitation results.
