# Supplementary material for: APDS treatment in Germany: decision criteria, costs and contribution of leniolisib to outcomes
Source: Front Immunol. 2026 Aug 10;17:1842815. doi: 10.3389/fimmu.2026.1842815 (PMC13507936; doi:10.3389/fimmu.2026.1842815)
Supplement: Supplementary file 1 [file DataSheet1.pdf]

PART 1: Standard of Care

Instructions for PART 1: Standard of Care

- Please provide your responses based on your experience in Germany
- Unless otherwise specified in the question, please provide answers considering APDS patients aged 12 and over only
- For a selection of manifestations (lymphoproliferation, cytopenia, gastrointestinal manifestations and advanced lung disease) the inverted Kaplan-Meier data from the European Society for Immunodeficiencies (ESID) are presented in this exercise for your information
- In **PART 1** of this exercise, you are asked to provide your lower plausible limit (L) and upper plausible limit (U) for the occurrence of manifestations and mortality amongst individuals with activated PI3K delta syndrome (APDS) treated with current **STANDARD OF CARE** (i.e. excluding leniolisib)
- Please add your responses to **all** cells outlined in **orange**
  - Although all questions are mandatory, if you feel particularly uncertain about your answer to a question, for example, you feel you do not have sufficient experience in a given age group to provide an estimate you feel confident in, please place an 'X' in the corresponding box(es) provided on each slide
- When providing your lower plausible limit and upper plausible limit, please consider the following:

Lower plausible limit (L):

You judge it to be extremely unlikely that the true value could be less than L should not be physically impossible, but extremely unlikely

Upper plausible limit (U):

You judge it to be extremely unlikely that the true value could be greater than U This This should not be physically impossible, but extremely unlikely

Abbreviations: APDS: Activated PI3K delta syndrome; ESID: European Society for Immunodeficiencies.

Respiratory Infections

- In the table below, please provide your lower and upper plausible estimates for the number of respiratory infections you would expect an APDS patient to experience per year, on standard of care

	Number of respiratory infections an APDS patient would experience per year	(desk research assumption:)
Lower plausible limit (L)	x	2
Upper plausible limit (U)	x	5
Please put an 'X' in the box if you feel particularly uncertain about answering the question	☐	

Abbreviations: APDS: Activated PI3K delta syndrome.

Other Types of Infection

- In the table below, please provide your lower and upper plausible estimates for the number of **other** types of infection you would expect an APDS patient to experience per year, on standard of care
- 'Other types of infection' include *non-respiratory* bacterial, viral and fungal infections (which are **not** Epstein–Barr virus [EBV] or cytomegalovirus [CMV])

	Number of other types of infection an APDS patient would experience per year	(desk research assumption:)
**Lower plausible limit (L)**	x	2
**Upper plausible limit (U)**	x	6
**Please put an 'X' in the box if you feel particularly uncertain about answering the question**	☐	

Abbreviations: APDS: Activated PI3K delta syndrome; CMV: Cytomegalovirus; EBV: Epstein–Barr virus.

Lymphoproliferation

- In the table below, please provide your lower and upper plausible estimates for the % of APDS patients with a history of lymphoproliferation (including lymphadenopathy and splenomegaly) by age 12, 30 and 40, on standard of care

Inverted Kaplan-Meier data from ESID

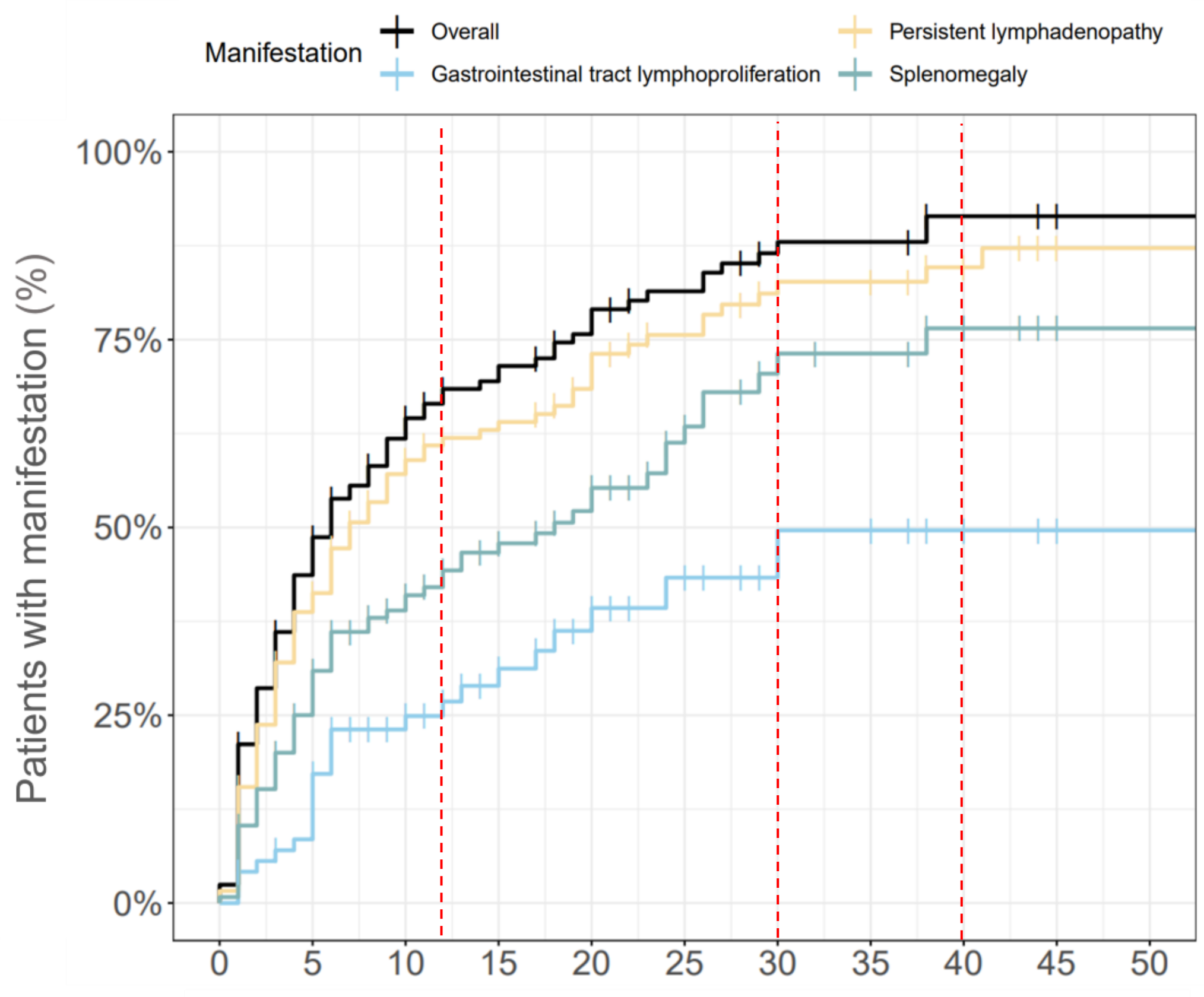

Kaplan-Meier data source: ESID

	% Of APDS patients with a history of lymphoproliferation		
	12 years	30 years	40 years
Estimates from ESID Kaplan-Meier data (‘**Overall**’ curve)	68.4%	88.0%	91.4%
Lower plausible limit (L)	x.x %	x.x %	x.x %
Upper plausible limit (U)	x.x %	x.x %	x.x %
Please put an ‘X’ in the box if you feel particularly uncertain about answering the question	☐	☐	☐

Footnote: Red dashed lines on the graph indicate ages at which data are presented in the table.
Abbreviations: APDS: Activated PI3K delta syndrome; ESID: European Society for Immunodeficiencies.

Cytopenia

- In the table below, please provide your lower and upper plausible estimates for the % of APDS patients with a history of autoimmune cytopenias (including neutropenia, anaemia and thrombocytopenia) by age 12, 25 and 40, on standard of care

Inverted Kaplan-Meier data from ESID

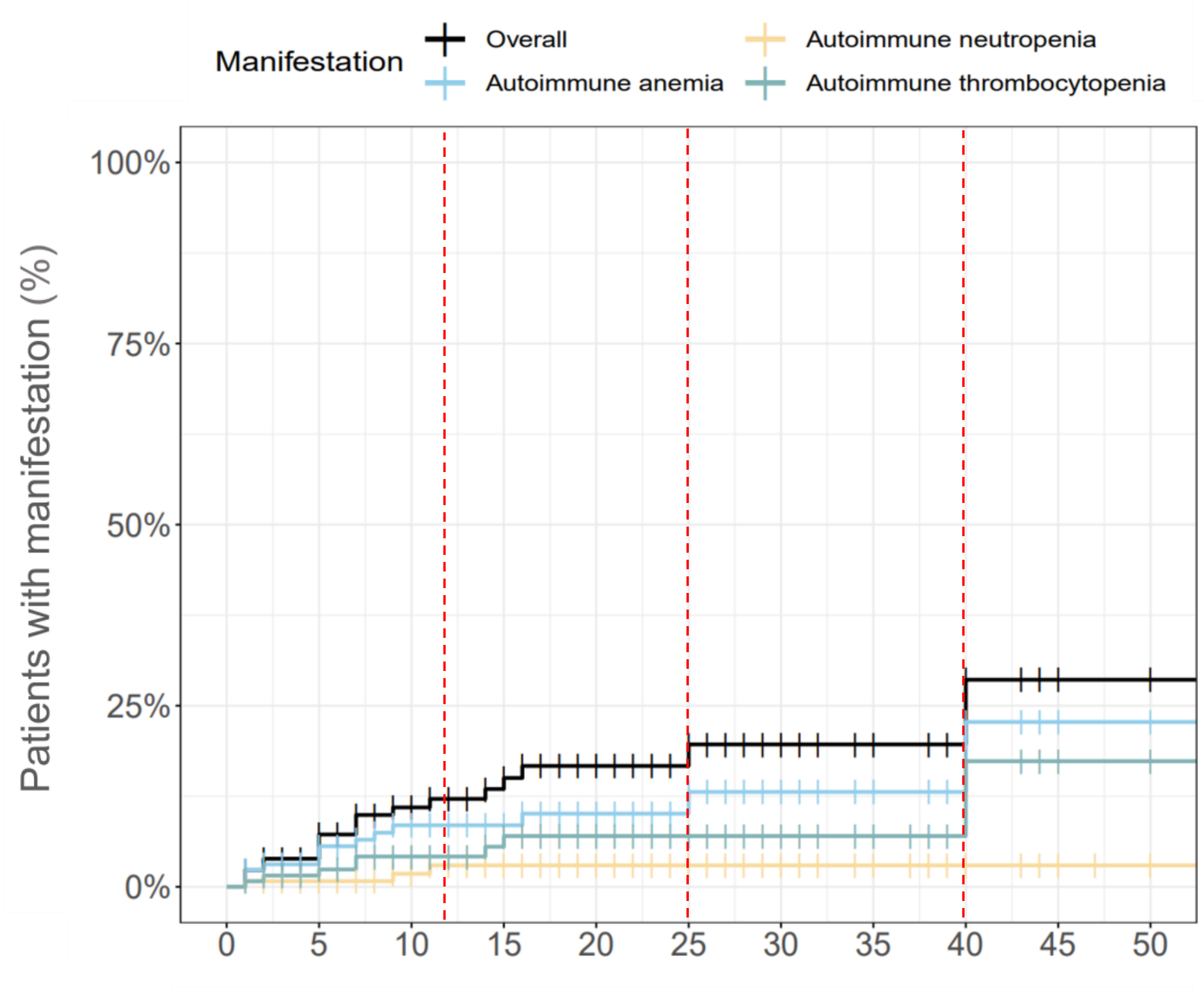

Kaplan-Meier data source: ESID

	% Of APDS patients with a history of autoimmune cytopenias		
	**12 years**	**25 years**	**40 years**
**Estimates from ESID Kaplan-Meier data ('Overall' curve)**	**12.1%**	**19.7%**	**28.6%**
**Lower plausible limit (L)**	x.x %	x.x %	x.x %
**Upper plausible limit (U)**	x.x %	x.x %	x.x %
**Please put an 'X' in the box if you feel particularly uncertain about answering the question**	☐	☐	☐

Footnote: Red dashed lines on the graph indicate ages at which data are presented in the table.
Abbreviations: APDS: Activated PI3K delta syndrome; ESID: European Society for Immunodeficiencies.

Gastrointestinal Manifestations

- In the table below, please provide your lower and upper plausible estimates for the % of APDS patients with a history of gastrointestinal (GI) manifestations by age 12, 25 and 35, on standard of care

Inverted Kaplan-Meier data from ESID

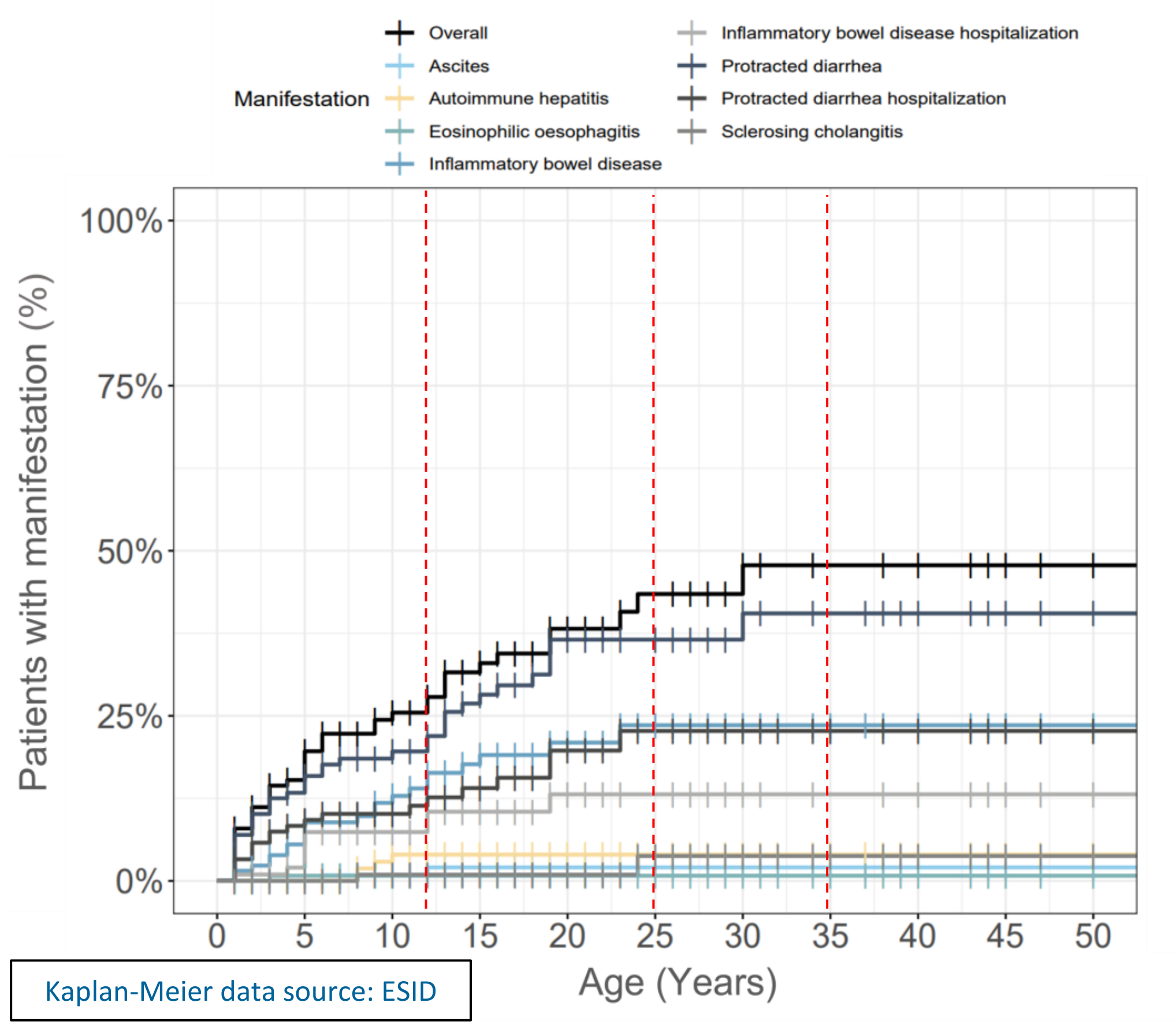

Kaplan-Meier data source: ESID

	% Of APDS patients with a history of GI manifestations		
	12 years	25 years	35 years
Estimates from ESID Kaplan-Meier data ('**Overall**' curve)	**27.9%**	**43.5%**	**47.8%**
**Lower plausible limit (L)**	x.x %	x.x %	x.x %
**Upper plausible limit (U)**	x.x %	x.x %	x.x %
**Please put an 'X' in the box if you feel particularly uncertain about answering the question**	☐	☐	☐

Footnote: Red dashed lines on the graph indicate ages at which data are presented in the table.
Abbreviations: APDS: Activated PI3K delta syndrome; ESID: European Society for Immunodeficiencies; GI: Gastrointestinal.

Bronchiectasis-Associated Airway Disease

- In the table below, please provide your lower and upper plausible estimates for the % of APDS patients with a history of bronchiectasis-associated airway disease by age 12, 30 and 40, on standard of care

Inverted Kaplan-Meier data from ESID

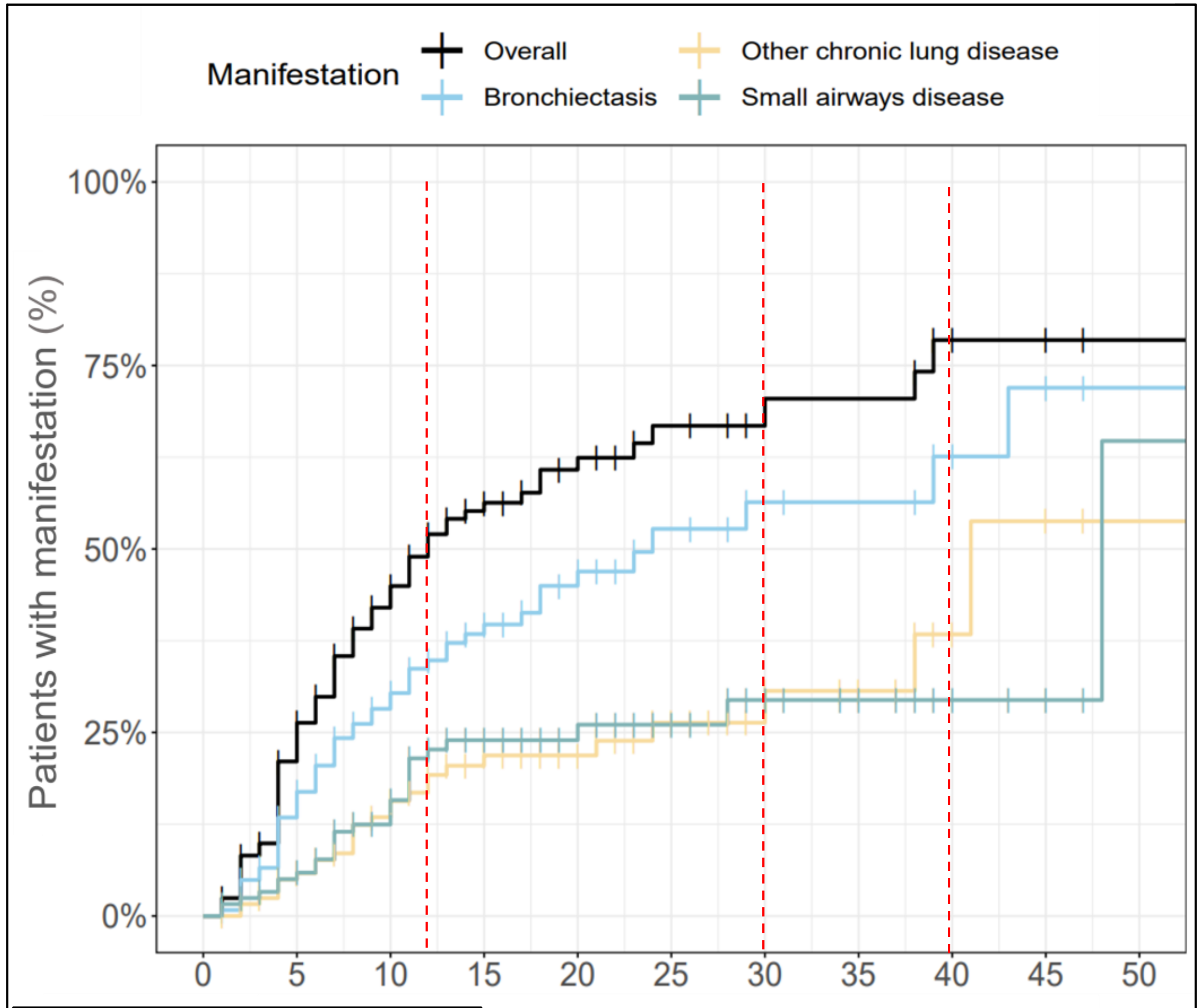

Kaplan-Meier data source: ESID

	% Of APDS patients with a history of bronchiectasis-associated airway disease		
	12 years	30 years	40 years
Estimates from ESID Kaplan-Meier data ('**Overall**' curve)	52.0%	70.5%	78.5%
Lower plausible limit (L)	x.x %	x.x %	x.x %
Upper plausible limit (U)	x.x %	x.x %	x.x %
Please put an 'X' in the box if you feel particularly uncertain about answering the question	☐	☐	☐

Footnote: Red dashed lines on the graph indicate ages at which data are presented in the table.
Abbreviations: APDS: Activated PI3K delta syndrome; ESID: European Society for Immunodeficiencies.

Advanced Lung Disease

- In the table below, please provide your lower and upper plausible estimates for the % of APDS patients with a history of advanced lung disease by age 12, 35 and 45, on standard of care. When answering this question, please consider advanced lung disease to be that which substantially impacts mortality and/or health-related quality of life (HRQoL). If you find it helpful, please feel free to use the data below to inform your answer

Inverted Kaplan-Meier data from ESID

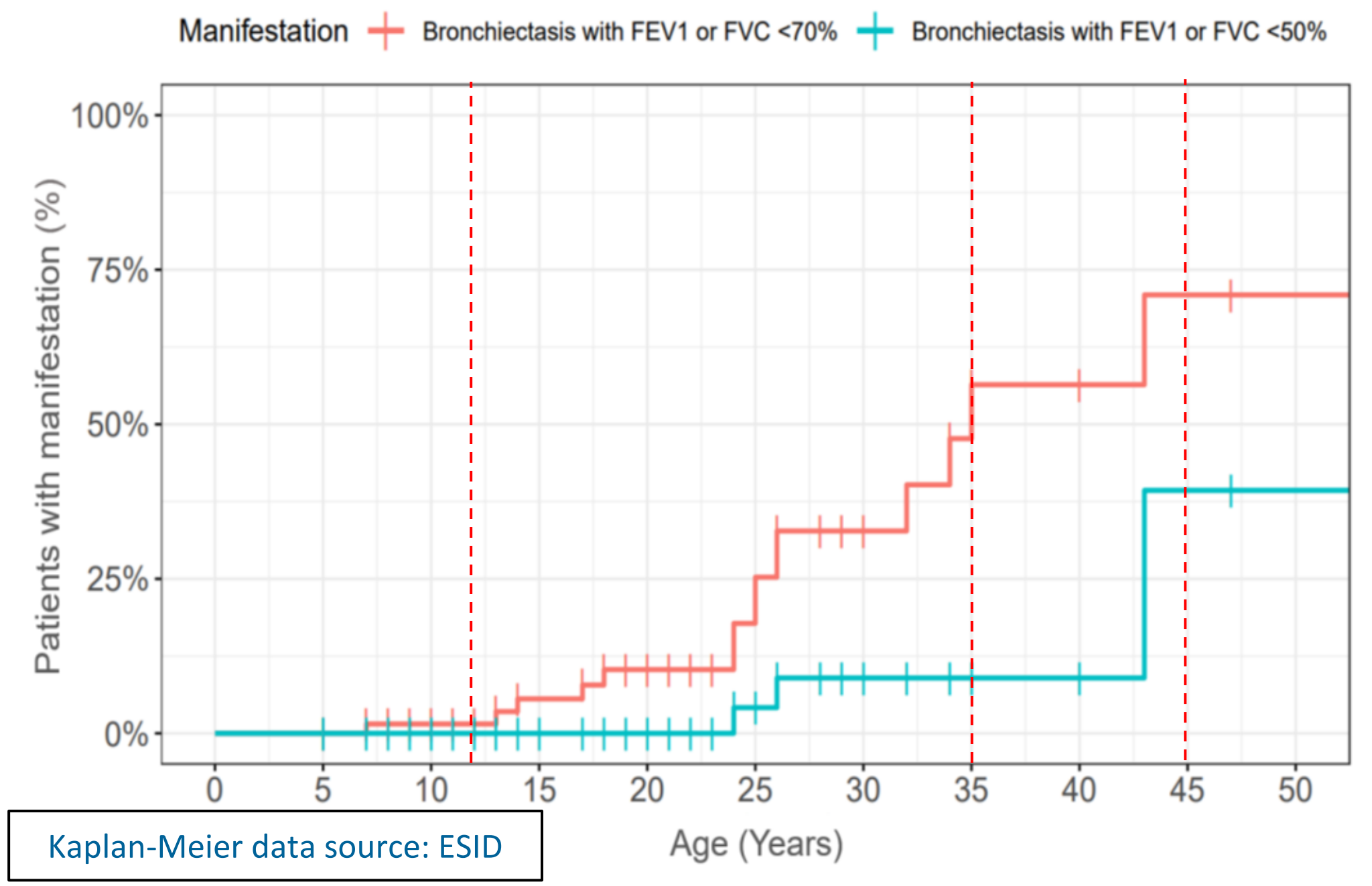

Kaplan-Meier data source: ESID

	% Of APDS patients with a history of advanced lung disease		
	12 years	35 years	45 years
Lower plausible limit (L)	x.x %	x.x %	x.x %
Upper plausible limit (U)	x.x %	x.x %	x.x %
Please put an 'X' in the box if you feel particularly uncertain about answering the question	☐	☐	☐

Footnote: FEV1 and FVC are % predicted values; Red dashed lines on the graph indicate ages at which data are presented in the table.
Abbreviations: APDS: Activated PI3K delta syndrome; FEV1: Forced expiratory volume 1; FVC: Forced vital capacity; HRQoL: Health-related quality of life.

Mortality

- In the table below, please provide your lower and upper plausible estimates for the % of APDS patients alive at age 12, 30 and 45, on standard of care

Kaplan-Meier data from Hanson et al. 2022

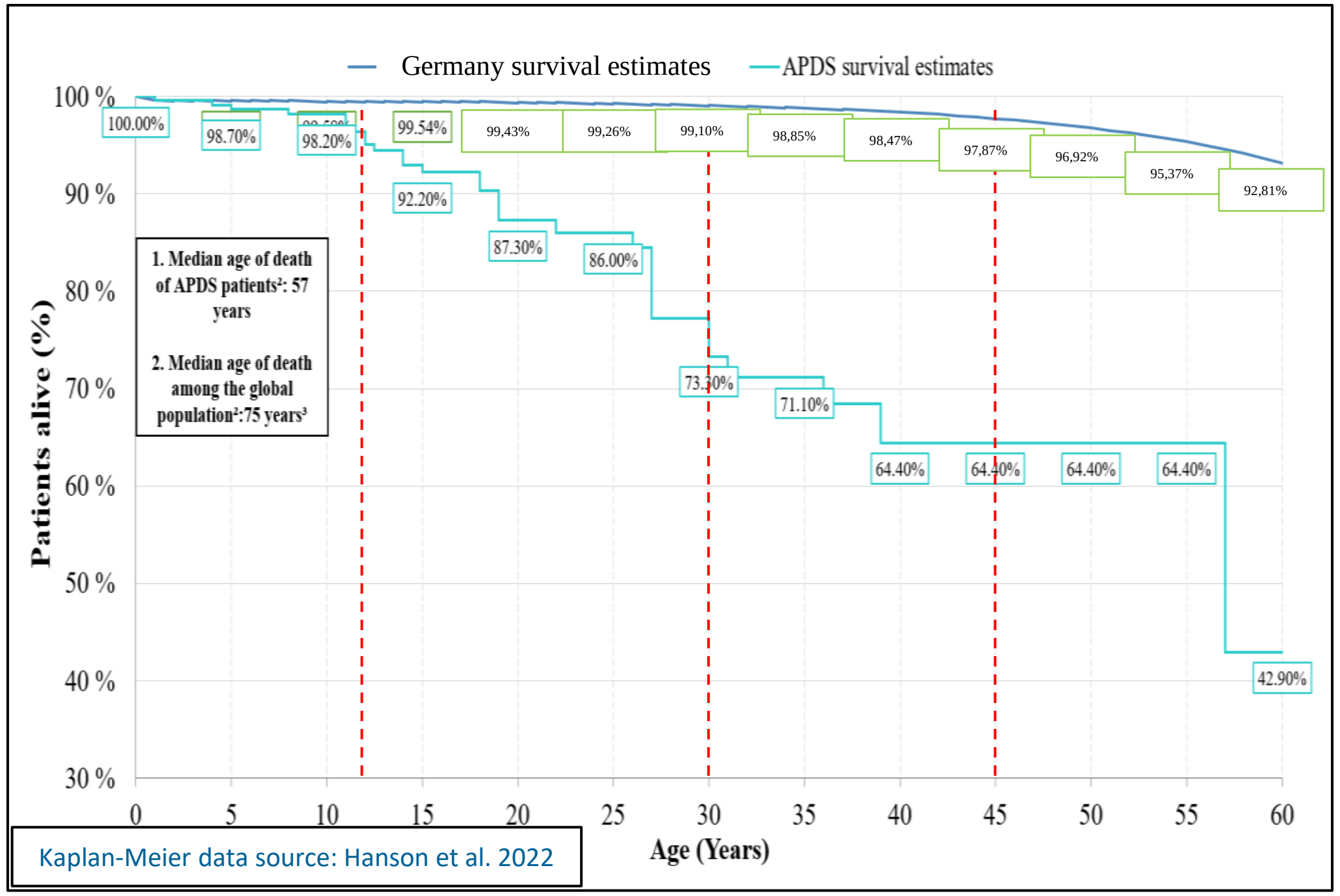

	% Of APDS patients alive		
	12 years	30 years	45 years
Estimates from Hanson 2022 (‘APDS survival estimates’ curve)	95.6%	73.3%	64.4%
Lower plausible limit (L)	x.x %	x.x %	x.x %
Upper plausible limit (U)	x.x %	x.x %	x.x %
Please put an ‘X’ in the box if you feel particularly uncertain about answering the question	☐	☐	☐

Footnote: Source of data for graph: Hanson et al. 2022; Red dashed lines on the graph indicate ages at which data are presented in the table.
Abbreviations: APDS: Activated PI3K delta syndrome.

Additional Comments for PART 1 “Standard of care”

- Please provide any comments in the box below

PART 2: Leniolisib

Instructions for PART 2: Leniolisib

- Please provide your responses based on your experience in Germany
- Unless otherwise specified in the question, please provide answers considering APDS patients aged 12 and over only
- Please add your responses to **<u>all</u>** cells outlined in **orange**
  - Although all questions are mandatory, if you feel particularly uncertain about your answer to a question, for example, you feel you do not have sufficient experience in a given age group to provide an estimate you feel confident in, please place an 'X' in the corresponding box(es) provided on each slide
- The aim of **PART 2** is to estimate the occurence of manifestations and mortality amongst individuals with APDS treated with **<u>LENIOLISIB</u>**
  - There are no published long-term data on the effects of leniolisib. However, health systems in Europe require information about the most likely long-term treatment effects
- In contrast to the standard of care, you should only provide plausible **point estimates** in part 2 (no lower and upper plausible limits)
- In the upcoming slides, you will be asked to provide predictions based on your **clinical expectations** for leniolisib:
  - In some cases, estimates are requested after APDS patients have been receiving leniolisib for a specific amount of time (e.g. 3 years). Please check the slide carefully for the assumed duration of leniolisib treatment in the question.

Abbreviations: APDS: Activated PI3K delta syndrome; ESID: European Society for Immunodeficiencies.

Respiratory Infections

- In the table below, please provide your plausible (point) estimate for the number of respiratory infections you would expect an APDS patient to experience per year when receiving leniolisib
- Please consider **APDS patients aged 12 and over** who have been **receiving leniolisib for 3 or more years** in your answer

	Number of respiratory infections you would expect an APDS patient to experience per year after receiving leniolisib for 3 or more years
Plausible (point) estimate	x
Please put an 'X' in the box if you feel particularly uncertain about answering the question	☐

Abbreviations: APDS: Activated PI3K delta syndrome.

Other Types of Infection

- In the table below, please provide your plausible (point) estimate for the number of **other** types of infection you would expect an APDS patient to experience per year when receiving leniolisib
- ‘Other types of infection’ include *non-respiratory* bacterial, viral and fungal infections (which are **not** Epstein–Barr virus [EBV] or cytomegalovirus [CMV])
- Please consider **APDS patients aged 12 and over** who have been **receiving leniolisib for 3 or more years** in your answer

	Number of other types of infection you would expect an APDS patient to experience per year after receiving leniolisib for 3 or more years
Plausible (point) estimate	x
Please put an ‘X’ in the box if you feel particularly uncertain about answering the question	☐

Abbreviations: APDS: Activated PI3K delta syndrome; CMV: Cytomegalovirus; EBV: Epstein–Barr virus.

Lymphoproliferation

- You will be asked two questions on lymphoproliferation across the next two slides:

Slide 1 (patients without lymphoproliferation [including lymphadenopathy and splenomegaly] at age 12):

- You are asked to assess the impact of leniolisib on the occurrence of lymphoproliferation:
  - In the table, please provide your plausible (point) estimate for the % of APDS patients developing lymphoproliferation 3 years (age 15) and 10 years (age 22) after initiating leniolisib

Slide 2 (patients with lymphoproliferation [including lymphadenopathy and splenomegaly]):

- You are asked to evaluate the impact of leniolisib on improving lymphoproliferation in patients aged 12 and over
  - In the table, please provide your plausible (point) estimate for the % of APDS patients that would experience **improvement** (with respect to lymphoproliferation [including lymphadenopathy and splenomegaly]) **after 3 years of receiving leniolisib**

Abbreviations: APDS: Activated PI3K delta syndrome; ESID: European Society for Immunodeficiencies; SoC: Standard of care.

Lymphoproliferation – Slide 1

- When answering this question:
  - Please consider **APDS patients without lymphoproliferation (including lymphadenopathy and splenomegaly) at age 12** who then begin leniolisib treatment

	% Of APDS patients without lymphoproliferation at age 12 who develop lymphoproliferation at age 15 and 22			
	3-year risk		10-year risk	
	SoC age 15	**Leniolisib age 15** (assume initiation of leniolisib at age 12)	SoC age 22	**Leniolisib age 22** (assume initiation of leniolisib at age 12)
**ESID point estimate**	9.7%	-	37.3%	-
**Plausible (point) estimate**		x.x %		x.x %
**Please put an 'X' in the box if you feel particularly uncertain about answering the question**		☐		☐

Footnote: Mean results from the previous round of this exercise shown in *italics*. Upper and lower estimates shifted to represent the population of interest. Please note zero participants felt particularly uncertain when answering this question in Part 1.

Abbreviations: APDS: Activated PI3K delta syndrome; ESID: European Society for Immunodeficiencies; SoC: Standard of care.

Explanation Charts: Lymphoproliferation

- % Of APDS patients without lymphoproliferation at age 12 who develop lymphoproliferation at age 15 and 22

SoC 3-year risk (age 12 to 15): 9.7%

Inverted Kaplan-Meier data from ESID

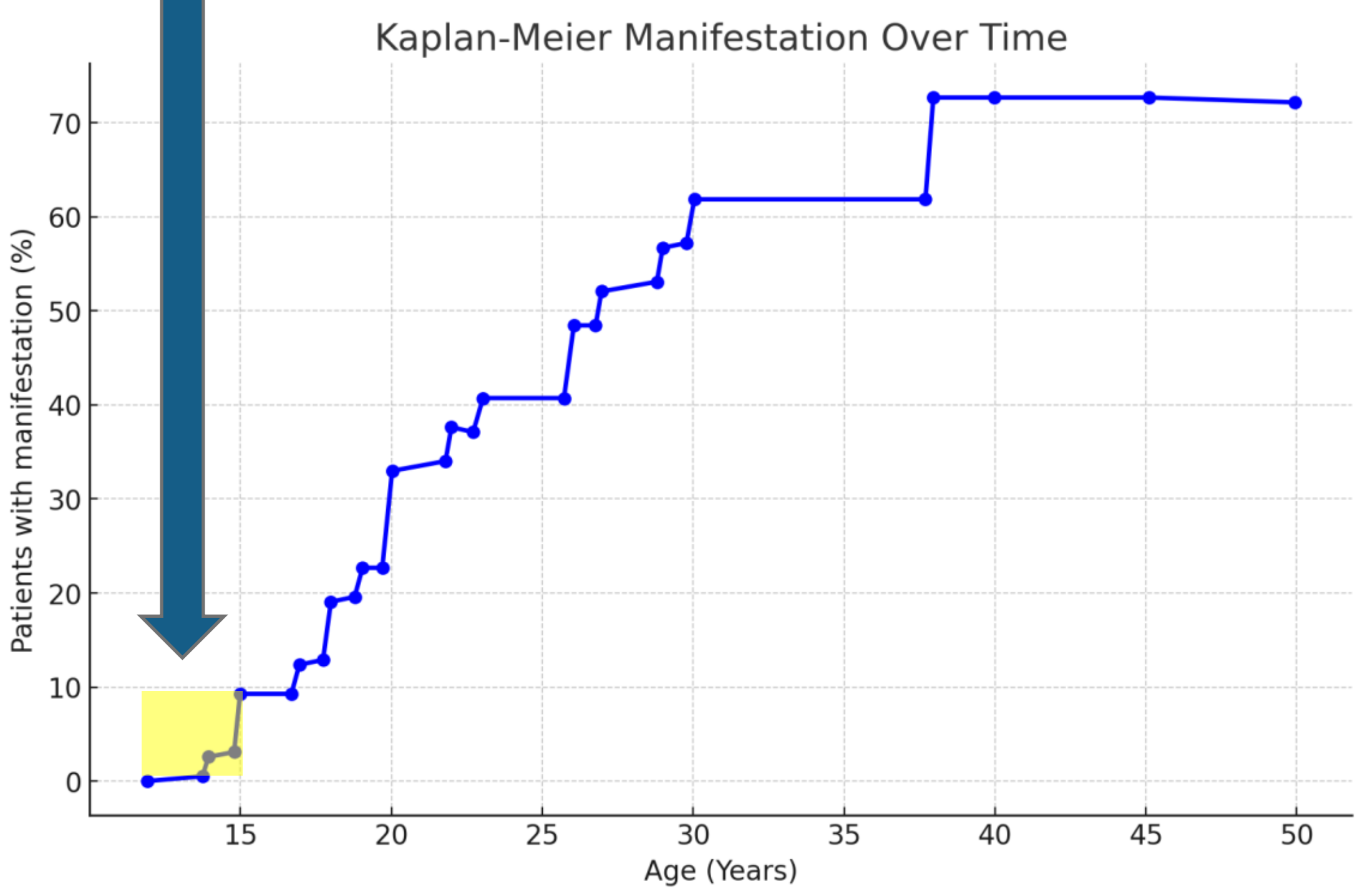

Kaplan-Meier data source: ESID

SoC 10-year risk (age 12 to 22): 37.3%

Inverted Kaplan-Meier data from ESID

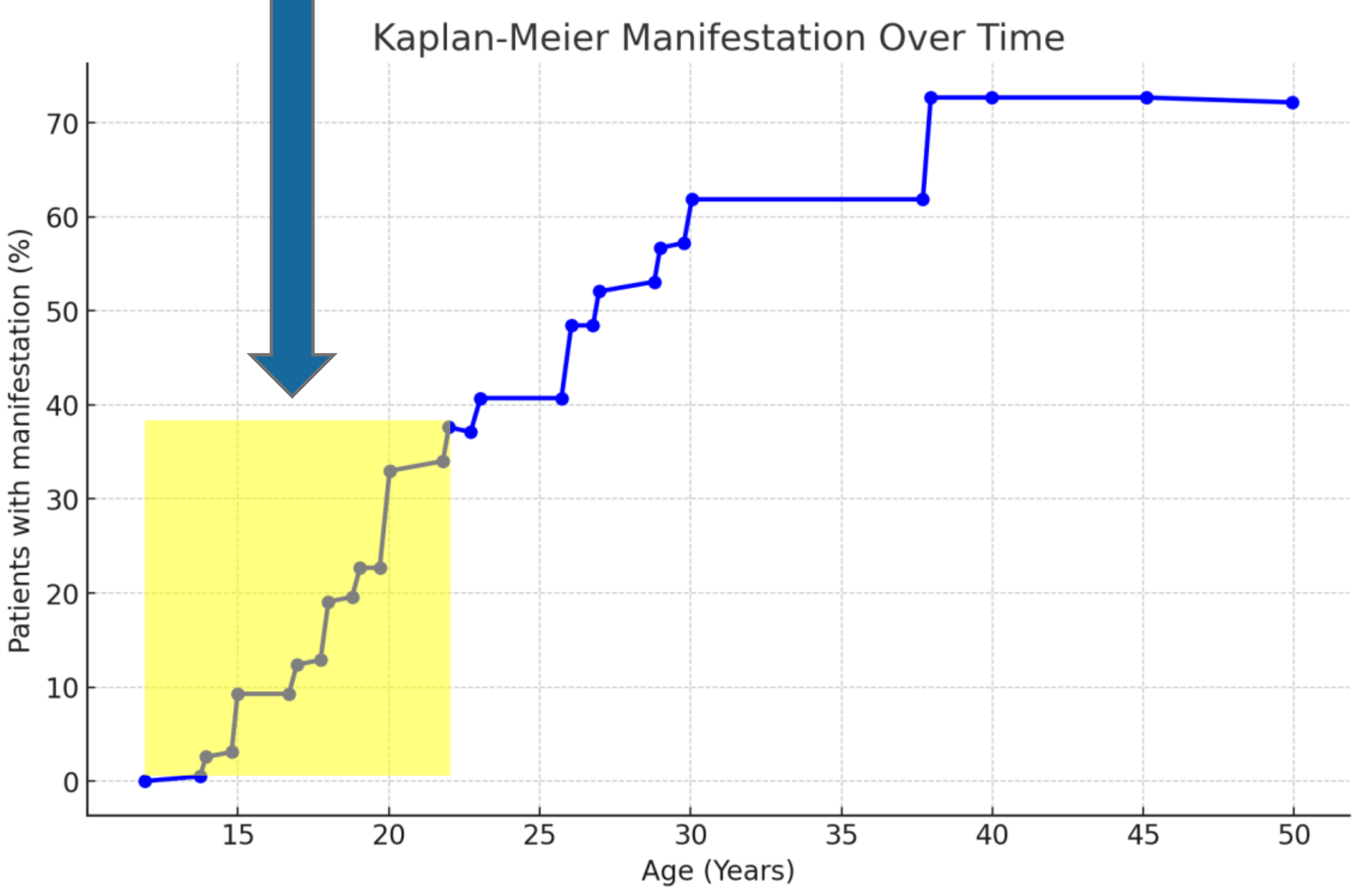

Kaplan-Meier data source: ESID

Lymphoproliferation – Slide 2

- In the table below, please provide a plausible (point) estimate for the percentage of patients aged 12 and over **with lymphoproliferation (including lymphadenopathy and splenomegaly)** who are expected to experience improvement after **3 years of receiving leniolisib**
- Consider "improvement" to be that which results in a substantial improvement in health-related quality of life

	**% Of APDS patients with lymphoproliferation that would experience improvement after 3 years of receiving leniolisib**
**Plausible (point) estimate**	x.x %
**Please put an 'X' in the box if you feel particularly uncertain about answering the question**	☐

Abbreviations: APDS: Activated PI3K delta syndrome; SoC: Standard of care.

Cytopenia

- You will be asked two questions on cytopenia across the next two slides:

Slide 1 (patients <u>without</u> cytopenia [autoimmune manifestations; including anaemia and thrombocytopenia] at age 12):

- You are asked to assess the impact of leniolisib on the occurrence of cytopenia:
  - In the table, please provide your plausible (point) estimate for the % of APDS patients developing cytopenia 3 years (age 15) and 10 years (age 22) after initiating leniolisib

Slide 2 (patients <u>with</u> cytopenia [autoimmune manifestations; including anaemia and thrombocytopenia]):

- You are asked to evaluate the impact of leniolisib on improving cytopenia in patients aged 12 and over
  - In the table, please provide your plausible (point) estimate for the % of APDS patients that would experience **improvement** (with respect to cytopenia [autoimmune manifestations; including anaemia and thrombocytopenia]) **after 3 years of receiving leniolisib**

Abbreviations: APDS: Activated PI3K delta syndrome; ESID: European Society for Immunodeficiencies; SoC: Standard of care.

Cytopenia – Slide 1

- When answering this question:
  - Please consider **APDS patients without cytopenia (autoimmune manifestations; including anaemia and thrombocytopenia) at age 12** who then begin leniolisib treatment

	% Of APDS patients without cytopenia at age 12 who develop cytopenia by age 15 and 22			
	3-year risk		10-year risk	
	SoC age 15	**Leniolisib age 15** (assume initiation of leniolisib at age 12)	SoC age 22	**Leniolisib age 22** (assume initiation of leniolisib at age 12)
**ESID point estimate**	3.3%	-	5.2%	-
**Plausible (point) estimate**		x.x %		x.x %
**Please put an 'X' in the box if you feel particularly uncertain about answering the question**		☐		☐

Footnote: Mean results from the previous round of this exercise shown in *italics*. Upper and lower estimates shifted to represent the population of interest.
Please note one participant felt particularly uncertain when answering this question in Part 1.

Abbreviations: APDS: Activated PI3K delta syndrome; ESID: European Society for Immunodeficiencies; SoC: Standard of care.

Explanation Charts: Cytopenia

- % Of APDS patients without cytopenia at age 12 who develop cytopenia by age 15 and 22

SoC 3-year risk (age 12 to 15): 3.3%
Inv ted Kaplan-Meier data from ESID

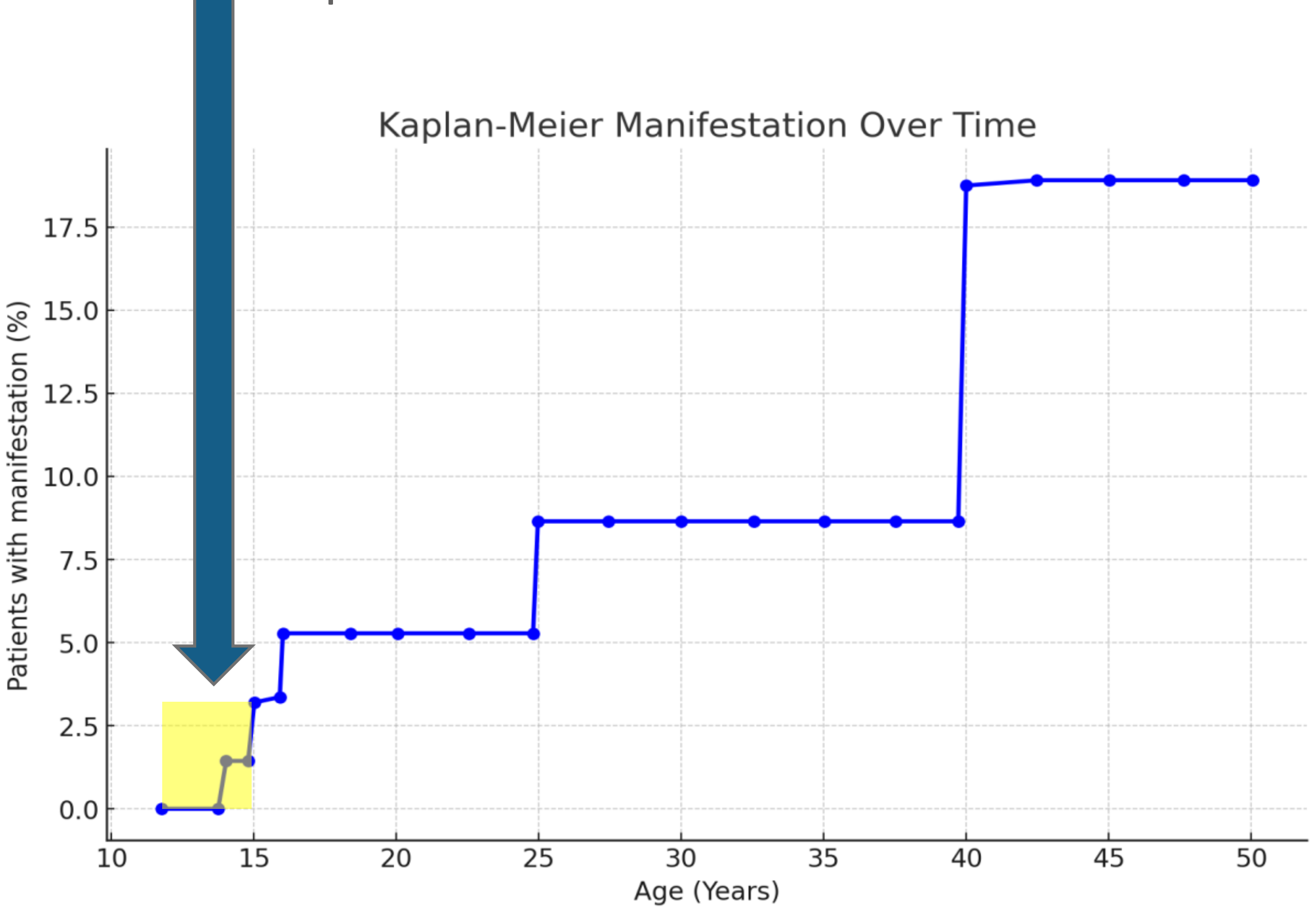

Kaplan-Meier data source: ESID

SoC 10-year risk (age 12 to 22): 5.2%
Inv ted Kaplan-Meier data from ESID

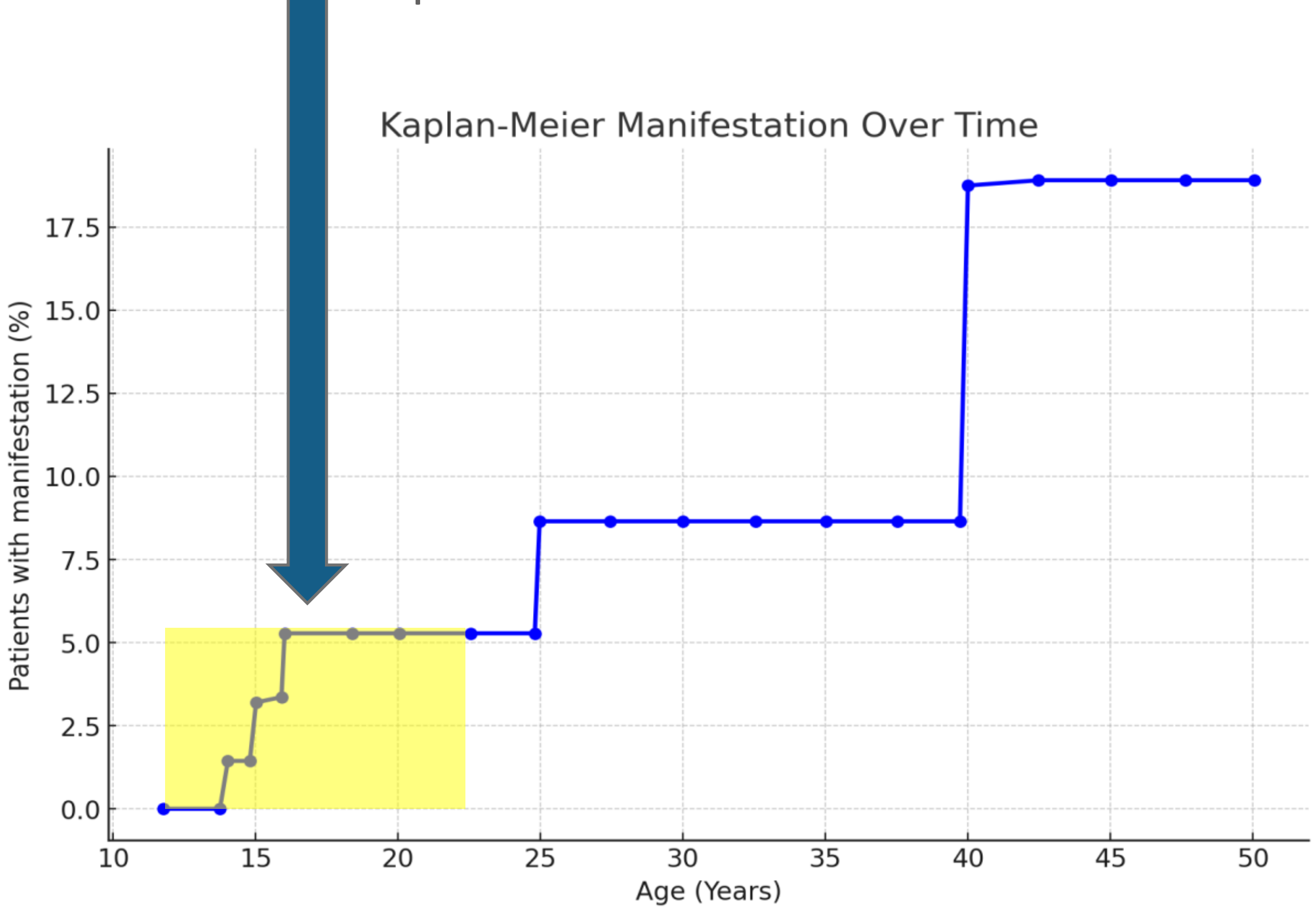

Kaplan-Meier data source: ESID

Cytopenia – Slide 2

- In the table below, please provide a plausible (point) estimate for the percentage of patients aged 12 and over **with cytopenia (autoimmune manifestations; including anaemia and thrombocytopenia)** who are expected to experience improvement **after 3 years of receiving leniolisib**
- Consider "improvement" to be that which results in a substantial improvement in health-related quality of life

	**% Of APDS patients with cytopenia that would experience improvement after 3 years of receiving leniolisib**
**Plausible (point) estimate**	x.x %
**Please put an 'X' in the box if you feel particularly uncertain about answering the question**	☐

Abbreviations: APDS: Activated PI3K delta syndrome; SoC: Standard of care.

Gastrointestinal Manifestations

- You will be asked two questions on gastrointestinal (GI) manifestations across the next two slides:

Slide 1 (patients without GI manifestations at age 12):

- You are asked to assess the impact of leniolisib on the occurrence of GI manifestations:
  - In the table, please provide your plausible (point) estimate for the % of APDS patients developing GI manifestations 3 years (age 15) and 10 years (age 22) after initiating leniolisib

Slide 2 (patients with GI manifestations):

- You are asked to evaluate the impact of leniolisib on improving GI manifestation symptoms in patients aged 12 and over
  - In the table, please provide your plausible (point) estimate for the % of APDS patients that would experience **improvement** (with respect to GI manifestation symptoms) **after 3 years of receiving leniolisib**

Abbreviations: APDS: Activated PI3K delta syndrome; ESID: European Society for Immunodeficiencies; GI: Gastrointestinal; SoC: Standard of care.

Gastrointestinal Manifestations – Slide 1

- When answering this question:
  - Please consider **APDS patients without GI manifestations at age 12** who then begin leniolisib treatment

	% Of APDS patients without GI manifestations at age 12 who develop GI manifestations by age 15 and 22			
	3-year risk		10-year risk	
	SoC age 15	Leniolisib age 15 (assume initiation of leniolisib at age 12)	SoC age 22	Leniolisib age 22 (assume initiation of leniolisib at age 12)
**ESID point estimate**	7.1%	-	14.3%	-
**Plausible (point) estimate**		x.x %		x.x %
**Please put an 'X' in the box if you feel particularly uncertain about answering the question**		☐		☐

Footnote: Mean results from the previous round of this exercise shown in *italics*. Upper and lower estimates shifted to represent the population of interest.
Please note zero participants felt particularly uncertain when answering this question in Part 1.

Abbreviations: APDS: Activated PI3K delta syndrome; ESID: European Society for Immunodeficiencies; GI: Gastrointestinal; SoC: Standard of care.

Explanation Charts: Gastrointestinal Manifestations

- % Of APDS patients without GI manifestations at age 12 who develop GI manifestations by age 15 and 22

SoC 3-year risk (age 12 to 15): 7.1%
Inverted Kaplan-Meier data from ESID

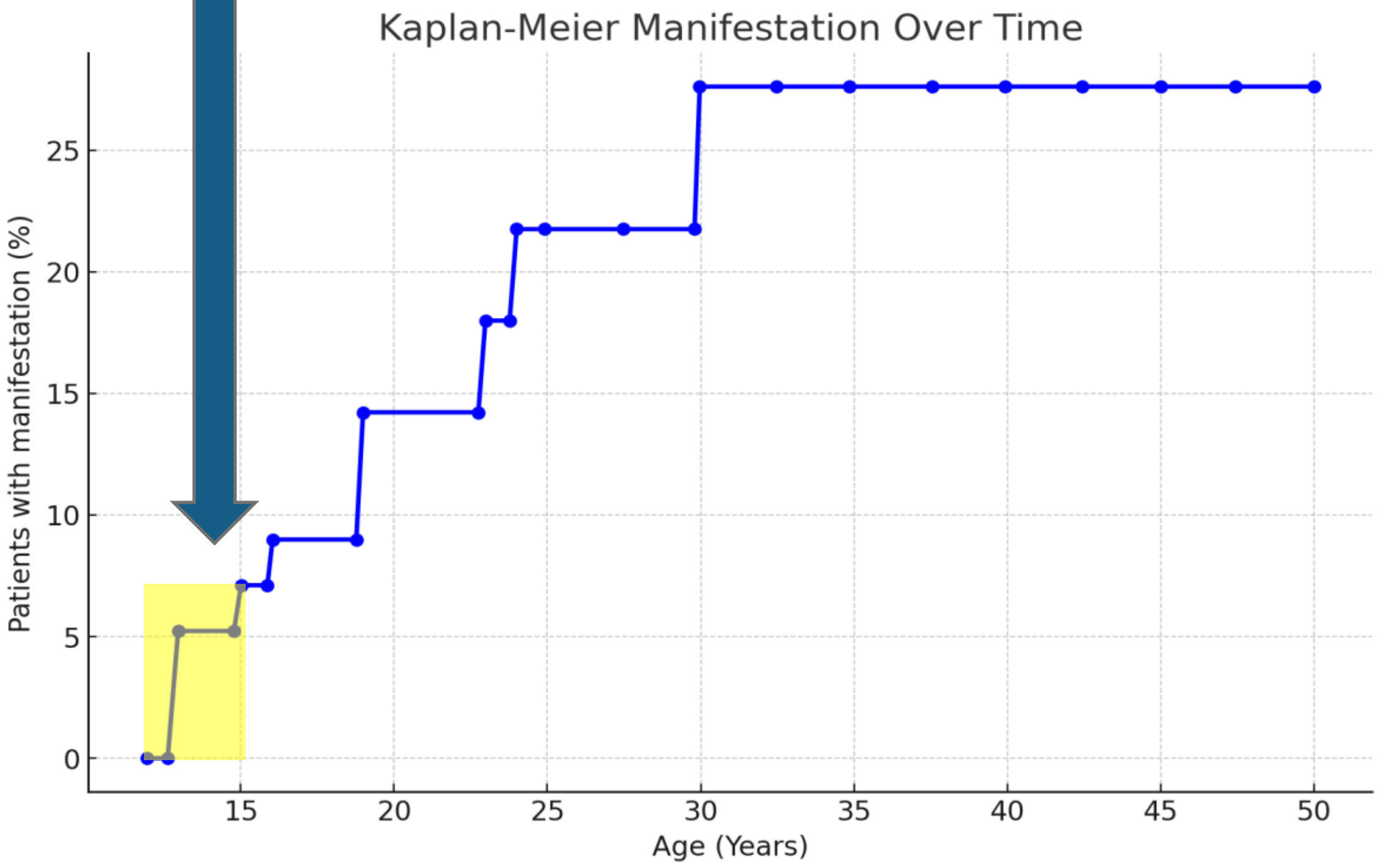

Kaplan-Meier data source: ESID

SoC 10-year risk (age 12 to 22): 14.3%
Inverted Kaplan-Meier data from ESID

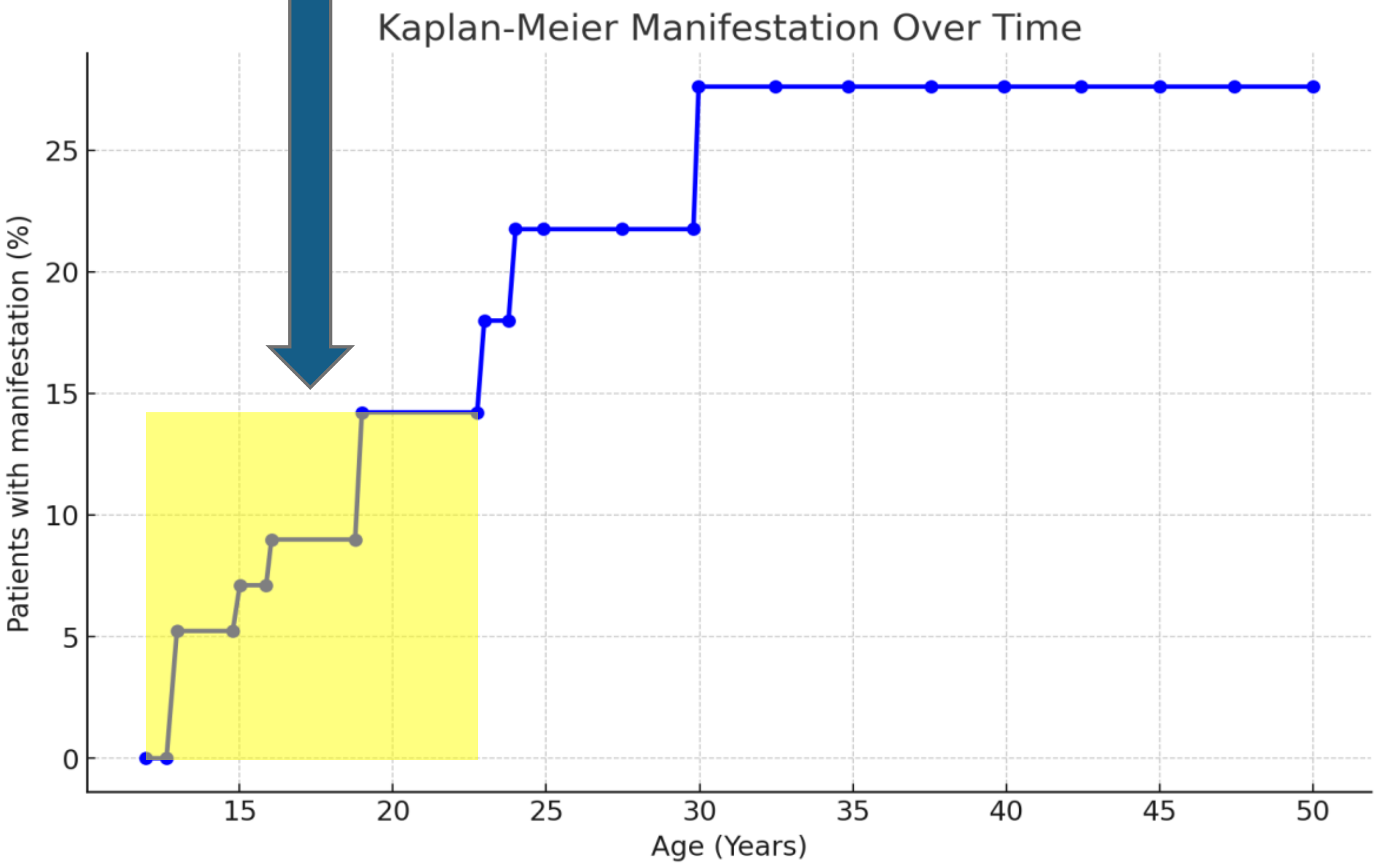

Kaplan-Meier data source: ESID

Gastrointestinal Manifestations – Slide 2

- In the table below, please provide a plausible (point) estimate for the percentage of patients aged 12 and over **with GI manifestations** who are expected to experience improvement **after 3 years of receiving leniolisib**
- Consider "improvement" to be that which results in a substantial improvement in health-related quality of life

	**% Of APDS patients with GI manifestations that would experience improvement after 3 years of receiving leniolisib**
**Plausible (point) estimate**	x.x %
**Please put an 'X' in the box if you feel particularly uncertain about answering the question**	☐

Abbreviations: APDS: Activated PI3K delta syndrome; GI: gastrointestinal; SoC: Standard of care.

Bronchiectasis-Associated Airway Disease

- You will be asked two questions on bronchiectasis-associated airway disease across the next two slides:

Slide 1 (patients without bronchiectasis-associated airway disease at age 12):

- You are asked to assess the impact of leniolisib on the occurrence of bronchiectasis-associated airway disease:
  - In the table, please provide your plausible (point) estimate for the % of APDS patients developing bronchiectasis-associated airway disease 3 years (age 15) and 10 years (age 22) after initiating leniolisib

Slide 2 (patients with bronchiectasis-associated airway disease):

- You are asked to evaluate the impact of leniolisib on improving bronchiectasis-associated airway disease in patients aged 12 and over
  - In the table, please provide your plausible (point) estimate for the % of APDS patients that would experience **improvement** (with respect to bronchiectasis-associated airway disease) **after 3 years of receiving leniolisib**

Abbreviations: APDS: Activated PI3K delta syndrome; ESID: European Society for Immunodeficiencies; SoC: Standard of care.

Bronchiectasis-Associated Airway Disease – Slide 1

- When answering this question:
  - Please consider **APDS patients without bronchiectasis-associated airway disease at age 12** who then begin leniolisib treatment

	% Of APDS patients without bronchiectasis-associated airway disease at age 12 who develop bronchiectasis-associated airway disease by age 15 and 22			
	3-year risk		10-year risk	
	SoC age 15	Leniolisib age 15 (assume initiation of leniolisib at age 12)	SoC age 22	Leniolisib age 22 (assume initiation of leniolisib at age 12)
ESID point estimate	8.9%	-	21.7%	-
Plausible (point) estimate		x.x %		x.x %
Please put an 'X' in the box if you feel particularly uncertain about answering the question		☐		☐

Footnote: Mean results from the previous round of this exercise shown in *italics*. Upper and lower estimates shifted to represent the population of interest.
Please note zero participants felt particularly uncertain when answering this question in Part 1.

Abbreviations: APDS: Activated PI3K delta syndrome; ESID: European Society for Immunodeficiencies; SoC: Standard of care.

Explanation Charts: Bronchiectasis-Associated Airway Disease

- % Of APDS patients without bronchiectasis-associated airway disease at age 12 who develop bronchiectasis-associated airway disease by age 15 and 22

SoC 3-year risk (age 12 to 15): 8.9%

Inverted Kaplan-Meier data from ESID

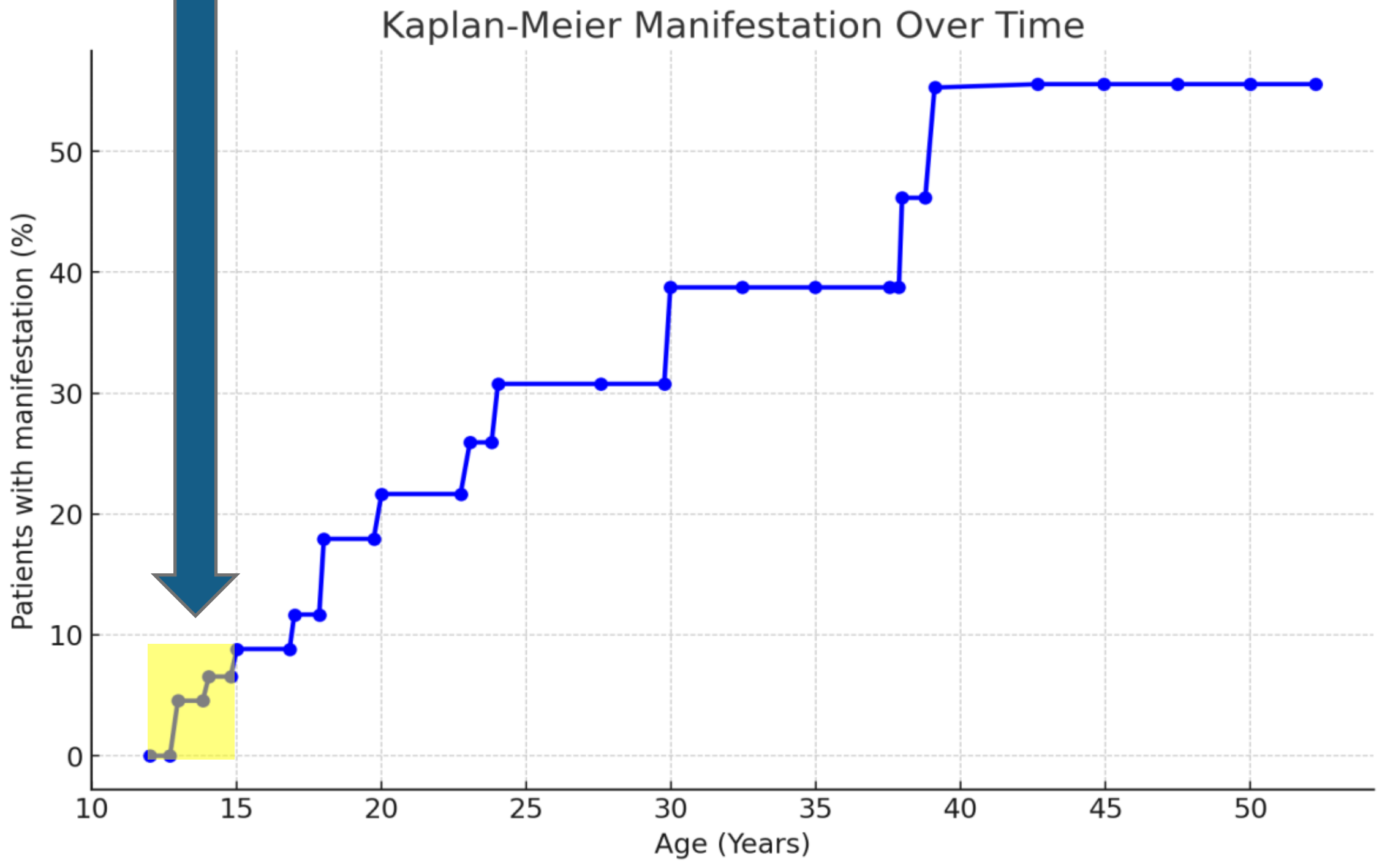

Kaplan-Meier data source: ESID

SoC 10-year risk (age 12 to 22): 21.7%

Inverted Kaplan-Meier data from ESID

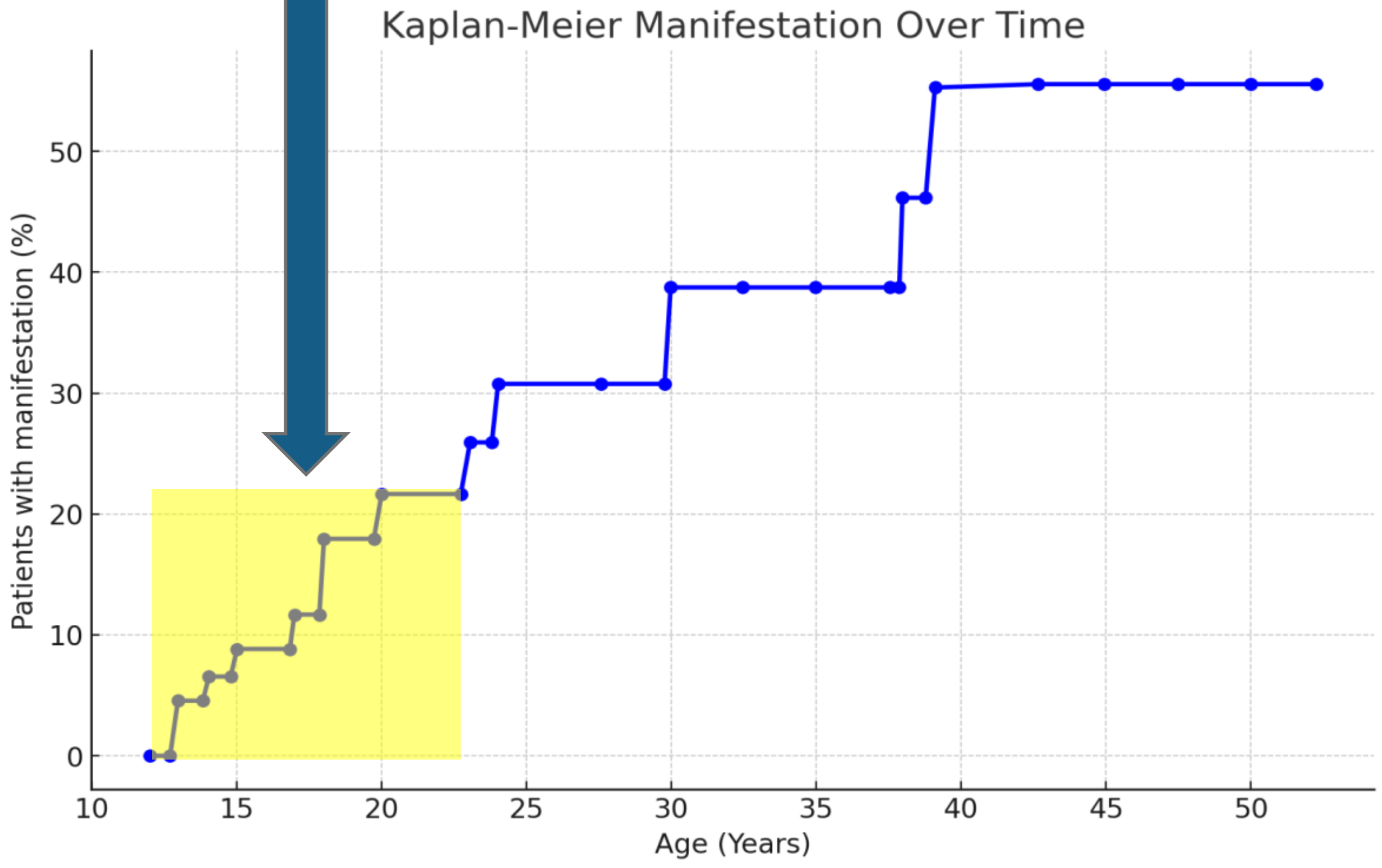

Kaplan-Meier data source: ESID

.

Bronchiectasis-Associated Airway Disease – Slide 2

- In the table below, please provide a plausible (point) estimate for the percentage of patients aged 12 and over **with bronchiectasis-associated airway disease** who are expected to experience improvement **after 3 years of receiving leniolisib**
- Consider "improvement" to be that which results in a substantial improvement in health-related quality of life

	**% Of APDS patients with bronchiectasis-associated airway disease that would experience improvement (with respect to bronchiectasis-associated airway disease) after 3 years of receiving leniolisib**
**Plausible (point) estimate**	x.x %
**Please put an 'X' in the box if you feel particularly uncertain about answering the question**	☐

**To what extent would you expect bronchiectasis-associated airway disease to improve in these APDS patients?**<br>*When answering this question, please consider measures of bronchiectasis severity including the bronchiectasis severity index (BSI), FACED score, % of Forced Expiratory Volume (FEV1), hospitalisations, exacerbations, modified Medical Research Council breathlessness score, colonisation of pseudomonas and other organisms, and radiological severity.*	

Abbreviations: APDS: Activated PI3K delta syndrome; BSI: Bronchiectasis severity index, ESID: European Society for Immunodeficiencies; FEV1: Forced Expiratory Volume; SoC: Standard of care.

Lymphoma

- You will be asked three questions on lymphoma across the next three slides:

Slide 1 (patients without lymphoma at age 12):

- You are asked to assess the impact of leniolisib on the occurrence of lymphoma:
  - In the table, please provide your plausible (point) estimate for the % of APDS patients developing lymphoma 3 years (age 15) and 10 years (age 22) after initiating leniolisib

Slide 2 (patients without lymphoma at age 20):

- You are asked to assess the impact of leniolisib on the occurrence of lymphoma:
  - In the table, please provide your plausible (point) estimate for the % of APDS patients developing lymphoma 3 years (age 23) and 10 years (age 30) after initiating leniolisib

Slide 3 (patients who have had and recovered from lymphoma by age 25):

- You are asked to evaluate the impact of leniolisib on the recurrence of lymphoma
  - In the table, In the table, please provide your lower and upper plausible estimates for the % of APDS patients aged 25 and over that you would expect to experience **recurrence** of lymphoma **within the following 10 years**, both on **SoC** and when receiving **leniolisib**

Abbreviations: APDS: Activated PI3K delta syndrome; ESID: European Society for Immunodeficiencies; SoC: Standard of care.

Lymphoma – Slide 1

- When answering this question:
  - Please consider **APDS patients without lymphoma at age 12** who then begin leniolisib treatment

	% Of APDS patients without lymphoma at age 12 who develop lymphoma by age 15 and 22			
	3-year risk		10-year risk	
	**SoC age 15**	**Leniolisib age 15** (assume initiation of leniolisib at age 12)	**SoC age 22**	**Leniolisib age 22** (assume initiation of leniolisib at age 12)
**ESID point estimate**	2.6%	-	10.5%	-
**Plausible (point) estimate**		x.x %		x.x %
**Please put an 'X' in the box if you feel particularly uncertain about answering the question**		☐		☐

Footnote: Mean results from the previous round of this exercise shown in *italics*. Upper and lower estimates shifted to represent the population of interest.
Please note one participant felt particularly uncertain when answering this question in Part 1.

Abbreviations: APDS: Activated PI3K delta syndrome; ESID: European Society for Immunodeficiencies; SoC: Standard of care.

Lymphoma – Slide 2

- When answering this question:
  - Please consider **APDS patients without lymphoma at age 20** who then begin leniolisib treatment

	% Of APDS patients without lymphoma at age 20 who develop lymphoma by age 23 and 30			
	3-year risk		10-year risk	
	SoC age 23	Leniolisib age 23 (assume initiation of leniolisib at age 20)	SoC age 30	Leniolisib age 30 (assume initiation of leniolisib at age 20)
**ESID point estimate**	0%	-	11.2%	-
**Plausible (point) estimate**		x.x %		x.x %
**Please put an 'X' in the box if you feel particularly uncertain about answering the question**		☐		☐

Footnote: Mean results from the previous round of this exercise shown in *italics*. Upper and lower estimates shifted to represent the population of interest.
Please note one participant felt particularly uncertain when answering this question in Part 1.

Abbreviations: APDS: Activated PI3K delta syndrome; ESID: European Society for Immunodeficiencies; SoC: Standard of care.

Lymphoma – Slide 3

- When answering this question:
  - Please consider **only those APDS patients who have had and recovered from lymphoma by age 25**
  - For the leniolisib estimates, please consider APDS patients who initiated leniolisib at age 25 in your answer

- In the table below, please provide your lower and upper plausible estimates for the % of APDS patients aged 25 and over that you would expect to experience recurrence of lymphoma within the following 10 years, both on **SoC** and when receiving **leniolisib**

	**In what % of APDS patients aged 25 years and over would lymphoma reoccur within 10 years post treatment initiation?**	
	**On SoC**	**On leniolisib**
**Lower plausible limit (L)**	x.x %	x.x %
**Upper plausible limit (U)**	x.x %	x.x %
**Please put an 'X' in the box if you feel particularly uncertain about answering the question**	☐	☐

Abbreviations: APDS: Activated PI3K delta syndrome; SoC: Standard of care.

Advanced Lung Disease

- You will be asked three questions on advanced lung disease across the next three slides:
- When answering these questions, please consider advanced lung disease to be defined as FEV1/FVC <70%

Slide 1 (patients without advanced lung disease at age 12):

- You are asked to assess the impact of leniolisib on the occurrence of advanced lung disease:
  - In the table, please provide your plausible (point) estimate for the % of APDS patients developing advanced lung disease 3 years (age 15) and 10 years (age 22) after initiating leniolisib

Slide 2 (patients without advanced lung disease at age 20):

- You are asked to assess the impact of leniolisib on the occurrence of advanced lung disease:
  - In the table, please provide your plausible (point) estimate for the % of APDS patients developing advanced lung disease 3 years (age 23) and 10 years (age 30) after initiating leniolisib

Slide 3 (patients with advanced lung disease):

- You are asked to evaluate the impact of leniolisib on improving advanced lung disease in patients aged 12 and over
  - In the table, please provide your plausible (point) estimate for the % of APDS patients that would experience **improvement** in advanced lung disease **after 3 years of receiving leniolisib**

Abbreviations: APDS: Activated PI3K delta syndrome; ESID: European Society for Immunodeficiencies; FEV1: forced expiratory volume 1; FVC: forced vital capacity; SoC: Standard of Care.

Advanced Lung Disease – Slide 1

- When answering this question:
  - Please consider **APDS patients without advanced lung disease (FEV1/FVC <70%) at age 12** who then begin leniolisib treatment

	% Of APDS patients without advanced lung disease at age 12 who develop advanced lung disease by age 15 and 22			
	3-year risk		10-year risk	
	SoC age 15	Leniolisib age 15 (assume initiation of leniolisib at age 12)	SoC age 22	Leniolisib age 22 (assume initiation of leniolisib at age 12)
ESID point estimate (FEV1 or FVC <70%)	4.1%	-	8.9%	-
Plausible (point) estimate		x.x %		x.x %
Please put an 'X' in the box if you feel particularly uncertain about answering the question		☐		☐

Footnote: Mean results from the previous round of this exercise shown in *italics*. Upper and lower estimates shifted to represent the population of interest.
Please note one participant felt particularly uncertain when answering this question in Part 1.

Abbreviations: APDS: Activated PI3K delta syndrome; ESID: European Society for Immunodeficiencies; FEV1: Forced Expiratory Volume 1; FVC: Forced Vital Capacity; SoC: Standard of care.

Explanation Charts: Advanced Lung Disease (FEV1/FVC <70%)

- % Of APDS patients without advanced lung disease at age 12 who develop advanced lung disease by age 15 and 22

SoC 3-year risk (age 12 to 15): 4.1%
Inverted Kaplan-Meier data from ESID

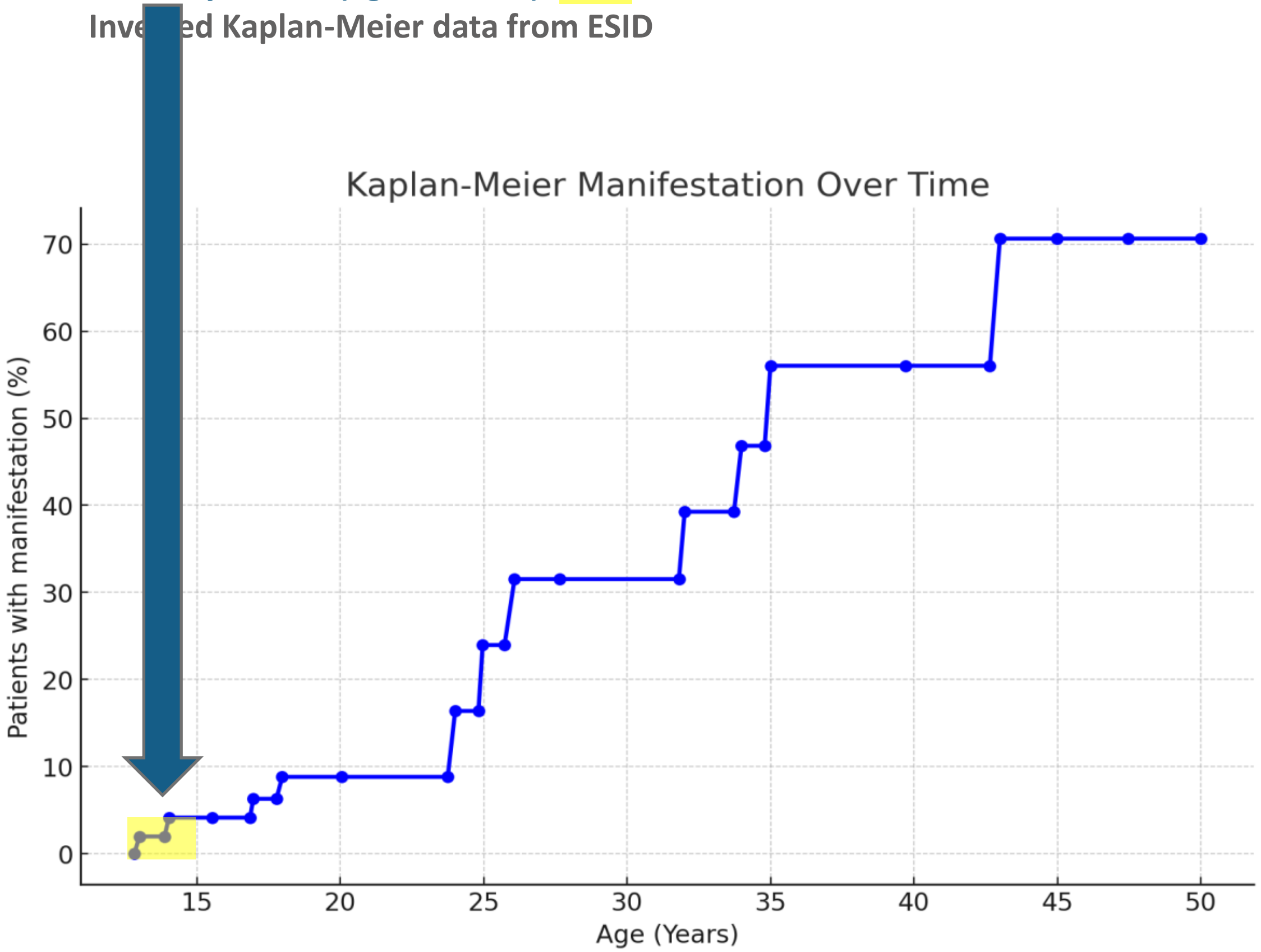

Kaplan-Meier data source: ESID

SoC 10-year risk (age 12 to 22): 8.9%
Inverted Kaplan-Meier data from ESID

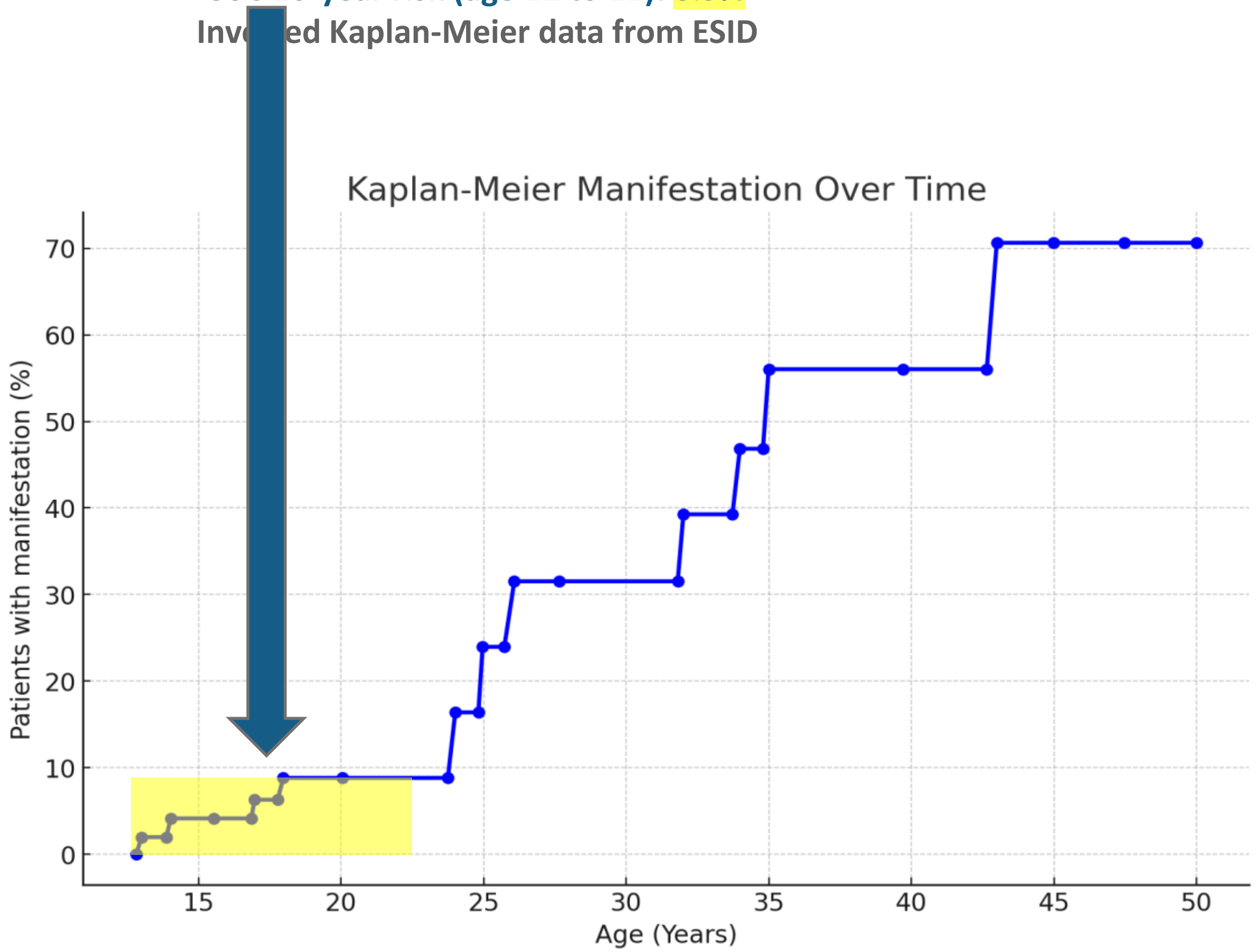

Kaplan-Meier data source: ESID

Advanced Lung Disease – Slide 2

- When answering this question:
  - Please consider **APDS patients without advanced lung disease (FEV1/FVC <70%) at age 20** who then begin leniolisib treatment

	% Of APDS patients without advanced lung disease at age 20 who develop advanced lung disease by age 23 and 30			
	**SoC age 23**	**Leniolisib age 23** (assume initiation of leniolisib at age 20)	**SoC age 30**	**Leniolisib age 30** (assume initiation of leniolisib at age 20)
**ESID point estimate (FEV1 or FVC <70%)**	0%	-	-	-
**Plausible (point) estimate**	*0%**	x.x %	*0%**	x.x %
**Please put an 'X' in the box if you feel particularly uncertain about answering the question**		☐		☐

Footnote: Mean results from the previous round of this exercise shown in *italics*. Upper and lower estimates shifted to represent the population of interest.
Please note one participant felt particularly uncertain when answering this question in Part 1.
*Please note, the exact lower plausible limit is -0.1%. It has been rounded to 0% to aid interpretation.
**Please note, the exact lower plausible limit is -27.4%. It has been rounded to 0% to aid interpretation.

Abbreviations: APDS: Activated PI3K delta syndrome; ESID: European Society for Immunodeficiencies; FEV1: Forced Expiratory Volume 1; FVC: Forced Vital Capacity; SoC: Standard of care.

Explanation Charts: Advanced Lung Disease (FEV1/FVC <70%)

- % Of APDS patients without advanced lung disease at age 20 who develop advanced lung disease by age 23

SoC 3-year risk (age 20 to 23): 0%

Inve[illegible]ed Kaplan-Meier data from ESID

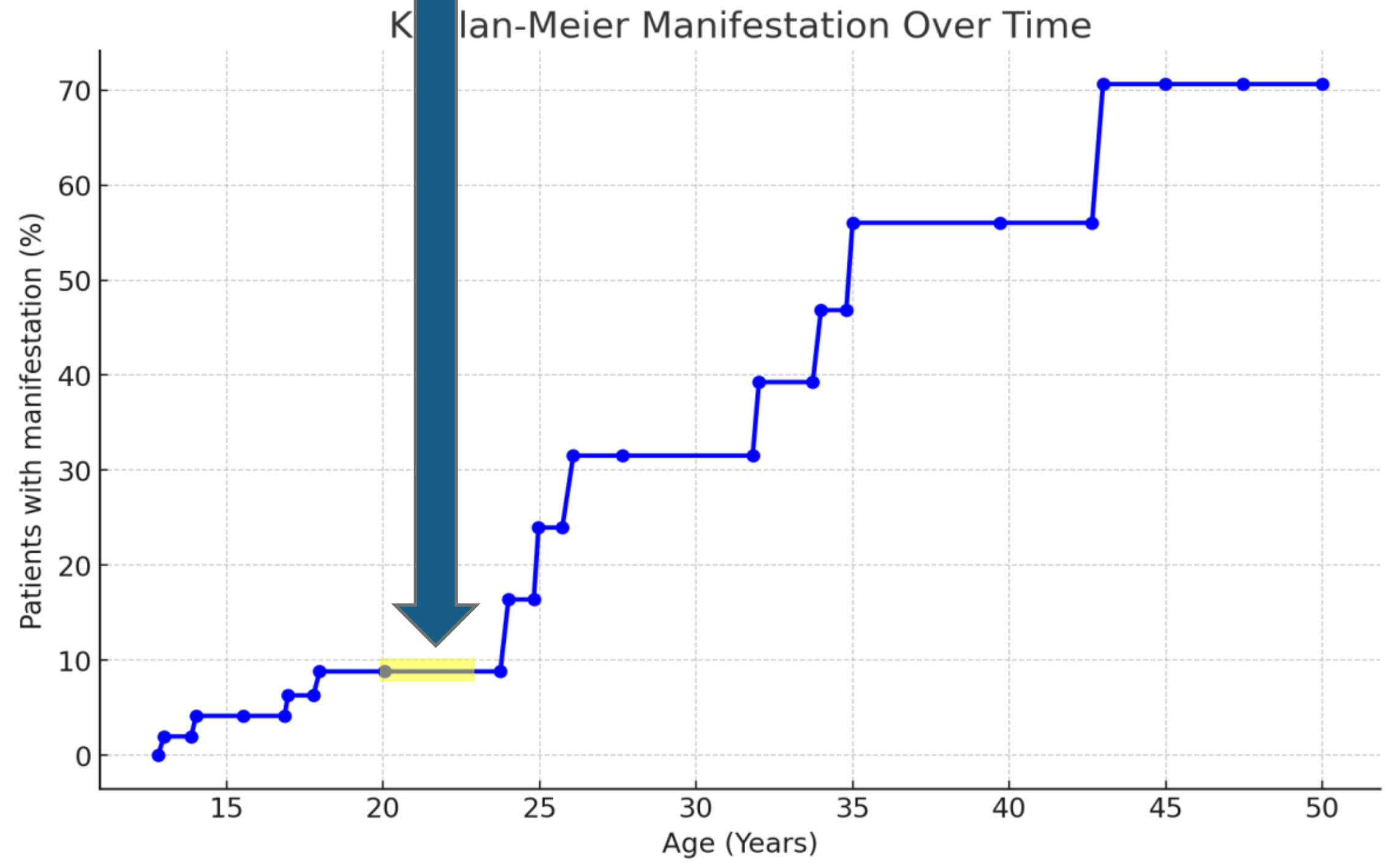

Kaplan-Meier data source: ESID

Advanced Lung Disease – Slide 3

- In the table below, please provide a plausible (point) estimate for the percentage of patients aged 12 and over **with advanced lung disease** who are expected to experience improvement after **3 years of receiving leniolisib**
- Consider "improvement" to be that which results in a substantial improvement in health-related quality of life

	**% Of APDS patients with advanced lung disease that would experience improvement after 3 years of receiving leniolisib**
**Plausible (point) estimate**	x.x %
**Please put an 'X' in the box if you feel particularly uncertain about answering the question**	☐

Abbreviations: APDS: Activated PI3K delta syndrome; SoC: Standard of care.

Mortality

- You are asked to assess the impact of leniolisib on **mortality**:
- In the table, please provide a plausible (point) estimate for the % of APDS patients that would be alive at age 20 and 40, assuming initiation of leniolisib at age 12

	% Of APDS patients alive at age 20 and 40			
	**SoC age 20**	**Leniolisib age 20** (assume initiation of leniolisib at age 12)	**SoC age 40**	**Leniolisib age 40** (assume initiation of leniolisib at age 12)
**Hanson et al. 2022 point estimate**	87.3%	-	64.4%	-
**Plausible (point) estimate**		x.x %		x.x %
**Please put an 'X' in the box if you feel particularly uncertain about answering the question**		☐		☐

Footnote: Mean results from the previous round of this exercise shown in *italics*. Upper and lower estimates shifted to represent the population of interest.
Please note one participant felt particularly uncertain when answering this question in Part 1.

Footnote: Source of data for Part 1 Results table: Hanson et al. 2022
Abbreviations: APDS: Activated PI3K delta syndrome; SoC: Standard of care.

Explanation Charts: Mortality

- % Of APDS patients alive at age 20 and 40

SoC patients alive at age 20: 87.3%
Kaplan-Meier data from Hanson et al. 2022

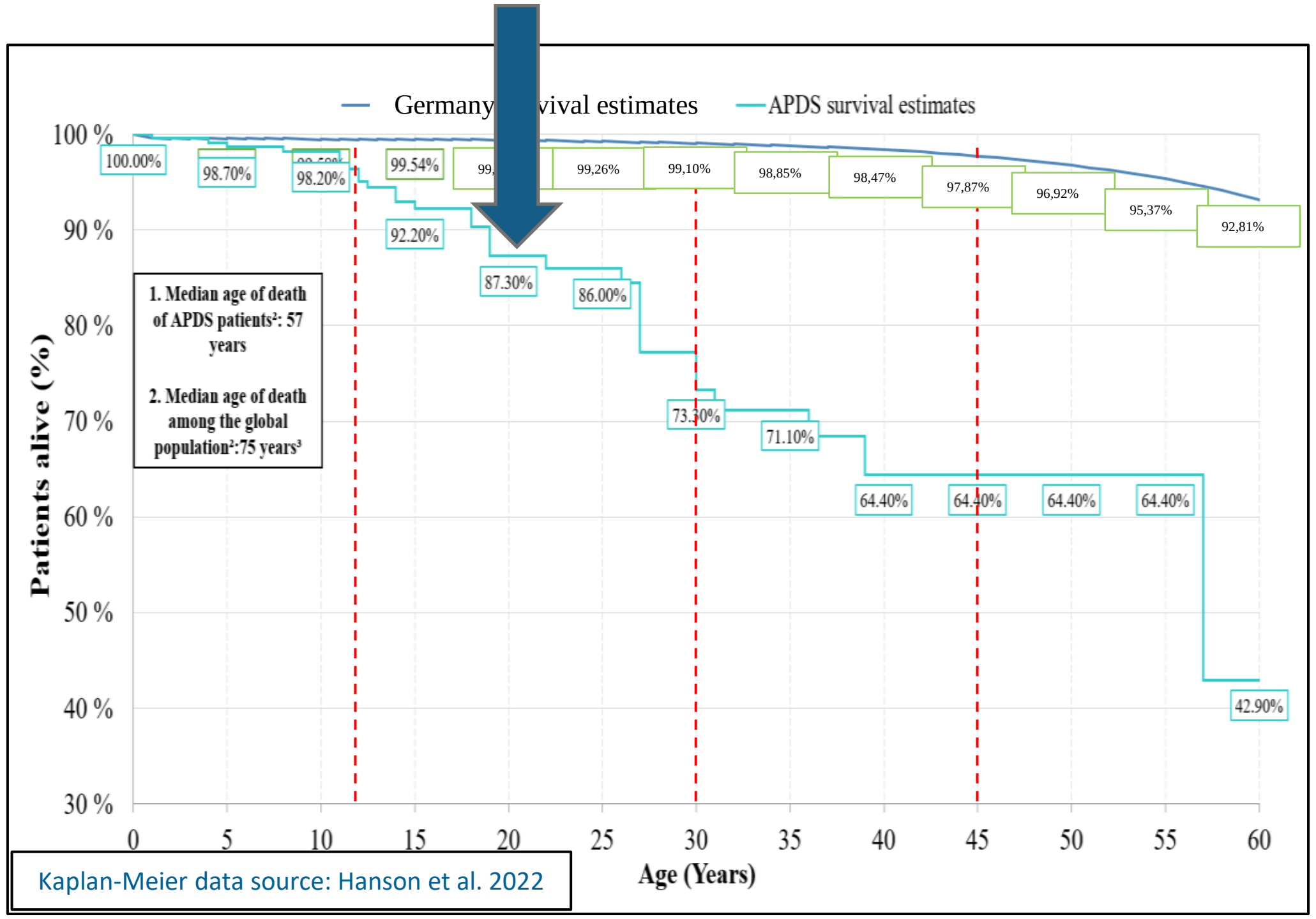

SoC patients alive at age 40: 64.4%
Kaplan-Meier data from Hanson et al. 2022

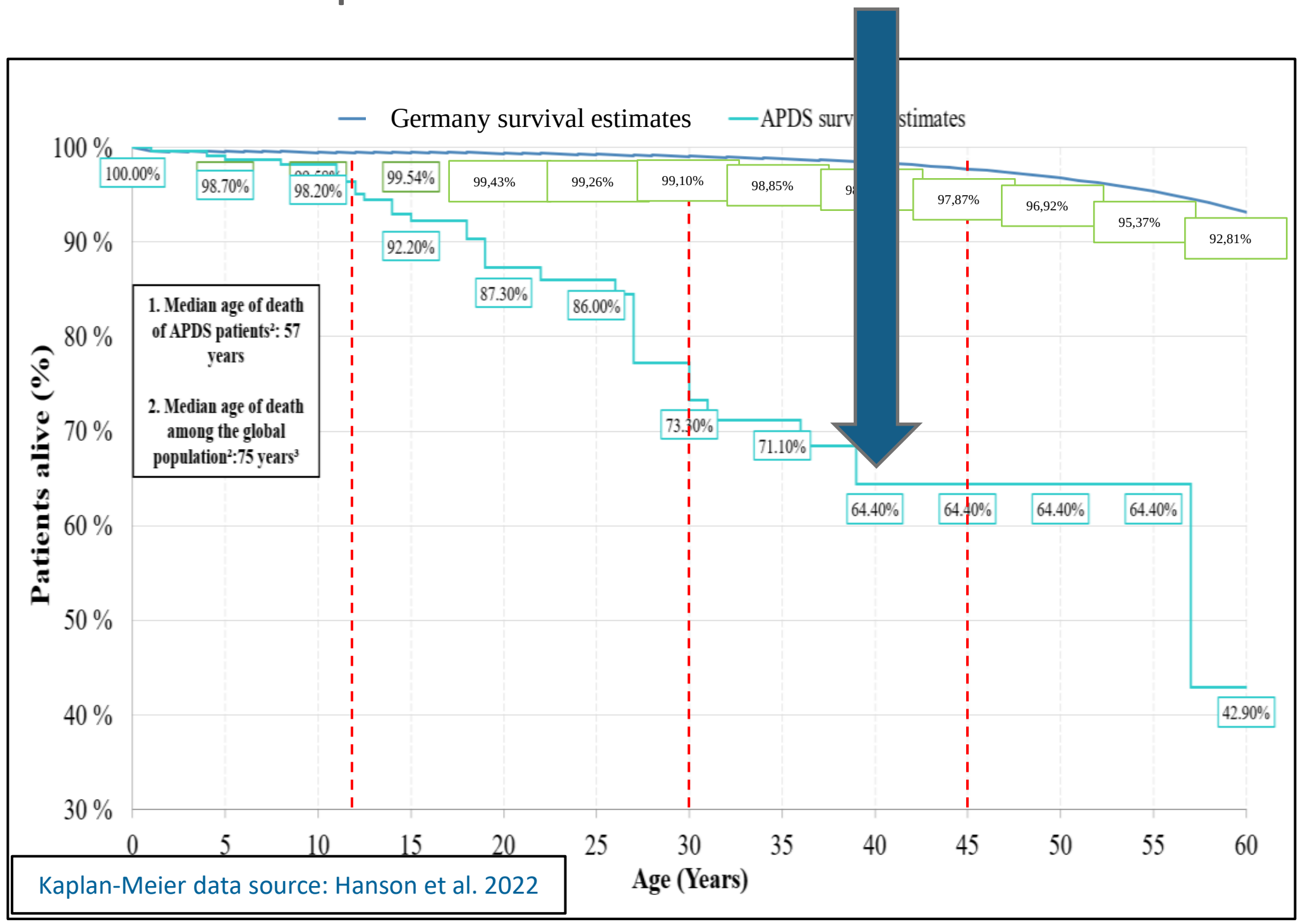

Footnote: Red dashed lines on the graph indicate ages at which data are presented in the table.
Abbreviations: APDS: Activated PI3K delta syndrome; ESID: European Society for Immunodeficiencies.

Additional Comments for PART 2: "Leniolisib"

- Please provide any comments in the box below
