# Supplementary material for: APDS treatment in Germany: decision criteria, costs and contribution of leniolisib to outcomes
Source: Front Immunol. 2026 Aug 10;17:1842815. doi: 10.3389/fimmu.2026.1842815 (PMC13507936; doi:10.3389/fimmu.2026.1842815)
Supplement: Supplementary file 2 [file DataSheet2.pdf]

ONLINE APPENDIX

Aggregated expert-elicitation results

Version: 15 July 2026

Panel: Six German clinical/scientific experts

Purpose. This appendix reports the anonymized aggregate results for all quantitative items from the expert-elicitation

Statistical note. Part 1 elicited lower and upper plausible limits for SoC; these are plausibility bounds and not confidence limits. Part 2 elicited point estimates for leniolisib, except the lymphoma-recurrence item after age 25, which elicited lower and upper plausible limits for both SoC and leniolisib. Missing responses were excluded item-wise. Percentages are displayed on a 0–100% scale.

Abbreviations: APDS, activated PI3K delta syndrome; CI, confidence interval; ESID, European Society for Immunodeficiencies; GI, gastrointestinal; L/U, lower/upper plausible limit; SD, standard deviation; SoC, standard of care.

Supplementary Table S1. Standard-of-care plausible limits

Endpoint	Estimate / time point	Unit	n	Mean	SD
Respiratory Infections	Lower L.	episodes/year	6	2.3	1.0
Respiratory Infections	Upper L.	episodes/year	6	8.0	1.3
Other Types of Infection	Lower L.	episodes/year	6	1.5	0.8
Other Types of Infection	Upper L.	episodes/year	6	5.3	1.2
Lymphoproliferation history, 12 years age	Lower L.	%	4	43.8	12.5
Lymphoproliferation history, 12 years age	Upper L.	%	4	78.2	2.4
Lymphoproliferation history, 30 years age	Lower L.	%	5	60.0	10.0
Lymphoproliferation history, 30 years age	Upper L.	%	5	87.0	7.6
Lymphoproliferation history, 40 years age	Lower L.	%	5	71.4	7.7
Lymphoproliferation history, 40 years age	Upper L.	%	5	90.4	6.6
Cytopenia history, 12 years age	Lower L.	%	5	7.9	3.1
Cytopenia history, 12 years age	Upper L.	%	5	23.0	2.7
Cytopenia history, 25 years age	Lower L.	%	6	15.0	4.5
Cytopenia history, 25 years age	Upper L.	%	6	30.0	4.5
Cytopenia history, 40 years age	Lower L.	%	6	21.5	6.6

Endpoint	Estimate / time point	Unit	n	Mean	SD
Cytopenia history, 40 years age	Upper L.	%	6	38.8	6.2
Gastrointestinal Manifestations 12 years	Lower L.	%	5	17.0	4.5
Gastrointestinal Manifestations 12 years	Upper L.	%	5	38.0	4.5
Gastrointestinal Manifestations 25 years	Lower L.	%	6	30.8	8.0
Gastrointestinal Manifestations 25 years	Upper L.	%	6	52.5	6.1
Gastrointestinal Manifestations 35 years	Lower L.	%	6	35.0	5.5
Gastrointestinal Manifestations 35 years	Upper L.	%	6	58.3	11.7
Bronchiectasis-Associated Airway Disease 12 years age	Lower L.	%	4	25.0	17.8
Bronchiectasis-Associated Airway Disease 12 years age	Upper L.	%	4	57.5	5.0
Bronchiectasis-Associated Airway Disease 30 years age	Lower L.	%	5	43.0	14.8
Bronchiectasis-Associated Airway Disease 30 years age	Upper L.	%	5	72.0	8.4
Bronchiectasis-Associated Airway Disease 40 years age	Lower L.	%	5	55.0	17.3
Bronchiectasis-Associated Airway Disease 40 years age	Upper L.	%	5	86.0	11.4
Advanced Lung Disease 12 years age	Lower L.	%	5	1.3	2.1
Advanced Lung Disease 12 years age	Upper L.	%	5	9.4	5.8
Advanced Lung Disease 35 years age	Lower L.	%	6	19.5	14.9
Advanced Lung Disease 35 years age	Upper L.	%	6	37.0	21.9
Advanced Lung Disease 45 years age	Lower L.	%	6	30.8	17.4
Advanced Lung Disease 45 years age	Upper L.	%	6	60.8	20.6
Survival: % alive at 12 years age	Lower L.	%	5	83.2	7.3
Survival: % alive at 12 years age	Upper L.	%	5	98.4	1.7
Survival: % alive at 30 years	Lower L.	%	6	61.5	8.2
Survival: % alive at 30 years	Upper L.	%	6	83.2	9.5
Survival: % alive at 45 years	Lower L.	%	6	47.5	13.3
Survival: % alive at 45 years	Upper L.	%	6	72.7	9.2

For Part 1, lower and upper plausible limits are summarized separately. They were not transformed into 95% confidence intervals.

Supplementary Table S2. Leniolisib estimates

Endpoint	Estimate / time point	Unit	n	Mean	SD
Respiratory Infections	Point estimate	episodes/year	6	2.5	0.8
Other Types of Infection	Point estimate	episodes/year	6	1.5	0.8
Lymphoproliferation: starting treatment at age 12 without pre-existing lymphoproliferation	3-year risk	%	6	7.3	5.4
Lymphoproliferation: starting treatment at age 12 without pre-existing lymphoproliferation	10-year risk	%	6	15.3	8.2
Lymphoproliferation: improvement if starting treatment at age 12 with pre-existing lymphoproliferation (including lymphadenopathy and splenomegaly)	3 years after Leniolisib	%	6	73.3	20.9
Cytopenia: starting treatment at age 12 without pre-existing cytopenia	3-year risk	%	6	2.2	2.0
Cytopenia: starting treatment at age 12 without pre-existing cytopenia	10-year risk	%	6	4.3	3.4
Cytopenia: improvement if starting treatment at age 12 with pre-existing cytopenia (autoimmune manifestations; including anaemia and thrombocytopenia)	3 years after Leniolisib	%	6	72.0	16.3
Gastrointestinal Manifestations: starting treatment at age 12 without pre-existing manifestation	3-year risk	%	6	5.4	1.6
Gastrointestinal Manifestations: starting treatment at age 12 without pre-existing manifestation	10-year risk	%	6	11.0	4.1
Gastrointestinal Manifestations: improvement if starting treatment at age 12 with pre-existing manifestation	3 years after Leniolisib	%	6	47.7	27.2
Bronchiectasis-Associated Airway Disease: starting treatment at age 12 without pre-existing manifestation	3-year risk	%	6	4.4	2.3
Bronchiectasis-Associated Airway Disease: starting treatment at age 12 without pre-existing manifestation	10-year risk	%	6	9.3	5.8
Bronchiectasis-Associated Airway Disease: improvement if starting treatment at age 12 with pre-existing manifestation	3 years after Leniolisib	%	5	50.0	38.6
Lymphoma Age 12: starting treatment at age 12 without pre-existing manifestation	3-year risk	%	6	1.5	0.9
Lymphoma Age 12: starting treatment at age 12 without pre-existing manifestation	10-year risk	%	6	4.4	3.4

Endpoint	Estimate / time point	Unit	n	Mean	SD
Lymphoma Age 20: starting treatment at age 20 without pre-existing manifestation	3-year risk	%	6	1.3	2.0
Lymphoma Age 20: starting treatment at age 20 without pre-existing manifestation	10-year risk	%	6	5.3	4.7
Lymphoma risk for recurrence: APDS patients who have had and recovered from lymphoma by age 25, SoC	10-year risk, Lower L.	%	6	9.0	7.4
Lymphoma risk for recurrence: APDS patients who have had and recovered from lymphoma by age 25, SoC	10-year risk, Upper L.	%	6	21.7	11.3
Lymphoma risk for recurrence: APDS patients who have had and recovered from lymphoma by age 25, with Leniolisib	10-year risk, Lower L.	%	6	3.2	3.8
Lymphoma risk for recurrence: APDS patients who have had and recovered from lymphoma by age 25, with Leniolisib	10-year risk, Upper L.	%	6	8.5	9.0
Advanced Lung Disease: starting treatment at age 12 without pre-existing manifestation	3-year risk	%	6	2.8	2.8
Advanced Lung Disease: starting treatment at age 12 without pre-existing manifestation	10-year risk	%	6	5.2	5.1
Advanced Lung Disease: starting treatment at age 20 without pre-existing manifestation	3-year risk	%	5	0.4	0.9
Advanced Lung Disease: starting treatment at age 20 without pre-existing manifestation	10-year risk	%	5	2.3	2.5
Advanced Lung Disease: improvement if starting treatment at age 12 with pre-existing advanced lung disease	3 years after Leniolisib	%	5	53.0	39.0
Survival: % alive at 20 years age (assume initiation of leniolisib at age 12)	Age 20 alive	%	6	94.8	3.8
Survival: % alive at 40 years age (assume initiation of leniolisib at age 12)	Age 40 alive	%	6	80.0	6.3

All values are descriptive aggregates. SD denotes sample standard deviation across the contributing expert responses.

Supplementary Table S3. SoC reference risks presented in the questionnaire

Comparator population	Horizon	SoC estimate (%)	Source shown to panel
Lymphoproliferation; no manifestation at age 12	3-year risk	9.7	ESID registry
Lymphoproliferation; no manifestation at age 12	10-year risk	37.3	ESID registry
Cytopenia; no manifestation at age 12	3-year risk	3.3	ESID registry
Cytopenia; no manifestation at age 12	10-year risk	5.2	ESID registry
Gastrointestinal manifestations; none at age 12	3-year risk	7.1	ESID registry
Gastrointestinal manifestations; none at age 12	10-year risk	14.3	ESID registry
Bronchiectasis-associated airway disease; none at age 12	3-year risk	8.9	ESID registry
Bronchiectasis-associated airway disease; none at age 12	10-year risk	21.7	ESID registry
Overt lymphoma; no lymphoma at age 12	3-year risk	2.6	ESID registry
Overt lymphoma; no lymphoma at age 12	10-year risk	10.5	ESID registry
Overt lymphoma; no lymphoma at age 20	3-year risk	0.0	ESID registry
Overt lymphoma; no lymphoma at age 20	10-year risk	11.2	ESID registry
Advanced lung disease; none at age 12	3-year risk	4.1	ESID registry
Advanced lung disease; none at age 12	10-year risk	8.9	ESID registry
Survival	Age 20	87.3	Hanson et al. 2022
Survival	Age 40	64.4	Hanson et al. 2022

These comparator values were presented to the panel as registry/literature anchors; they are not expert-derived point estimates and therefore do not have an expert-response SD.
